# *xopAC*-triggered Immunity against *Xanthomonas* Depends on *Arabidopsis* Receptor-Like Cytoplasmic Kinase Genes *PBL2* and *RIPK*

**DOI:** 10.1371/journal.pone.0073469

**Published:** 2013-08-09

**Authors:** Endrick Guy, Martine Lautier, Matthieu Chabannes, Brice Roux, Emmanuelle Lauber, Matthieu Arlat, Laurent D. Noël

**Affiliations:** 1 INRA, Laboratoire des Interactions Plantes Micro-organismes (LIPM), UMR 441, Castanet-Tolosan, France; 2 CNRS, Laboratoire des Interactions Plantes Micro-organismes (LIPM), UMR 2594, Castanet-Tolosan, France; 3 Université de Toulouse, Université Paul Sabatier, Toulouse, France; University of Wisconsin-Milwaukee, United States of America

## Abstract

*Xanthomonas campestris* pv. *campestris* (Xcc) colonizes the vascular system of Brassicaceae and ultimately causes black rot. In susceptible *Arabidopsis* plants, XopAC type III effector inhibits by uridylylation positive regulators of the PAMP-triggered immunity such as the receptor-like cytoplasmic kinases (RLCK) BIK1 and PBL1. In the resistant ecotype Col-0, *xopAC* is a major avirulence gene of *Xcc*. In this study, we show that both the RLCK interaction domain and the uridylyl transferase domain of XopAC are required for avirulence. Furthermore, *xopAC* can also confer avirulence to both the vascular pathogen *Ralstonia solanacearum* and the mesophyll-colonizing pathogen *Pseudomonas syringae* indicating that *xopAC*-specified effector-triggered immunity is not specific to the vascular system. *In planta*, XopAC-YFP fusions are localized at the plasma membrane suggesting that XopAC might interact with membrane-localized proteins. Eight RLCK of subfamily VII predicted to be localized at the plasma membrane and interacting with XopAC in yeast two-hybrid assays have been isolated. Within this subfamily, *PBL2* and *RIPK RLCK* genes but not *BIK1* are important for *xopAC*-specified effector-triggered immunity and *Arabidopsis* resistance to *Xcc*.

## Introduction

Plant innate immunity is a multilayer system which limits pathogen entry and multiplication in leaf tissues. These defence mechanisms are initially triggered by generic elicitors called PAMPs (Pathogen-associated molecular patterns) [1]. PAMPs are highly conserved, present in multiple organisms and are usually important for microbial fitness or viability [2]. For example, in bacteria, lipopolysaccharides, peptidoglycan, flagellin, AX21 secreted protein or the elongation factor-Tu (EF-Tu) rapidly elicit various defences such as plant cell wall reinforcement, ion fluxes, MAP kinase signalling cascades, transcriptional reprogramming or production of reactive oxygen species (ROS) and anti-bacterial compounds. These responses are globally referred to as PAMP-triggered immunity (PTI). Several pattern-recognition receptors (PRR) which specifically perceive the presence of the PAMPs have been identified at the plasma membrane [3]. The best-studied PRR are the receptor-like kinases (RLK) FLS2 (Flagellin sensing 2) and EFR (EF-Tu receptor) of *A. thaliana* which are required for flagellin/AX21 and EF-Tu perception, respectively [4–6]. Upon perception of flagellin and EF-Tu, both FLS2 and EFR dissociate from BIK1 ( *Botrytis* -induced kinase) and associate with the co-receptor BAK1 (BRI1-associated kinase) to initiate a highly complex phosphorylation cascade leading to the establishment of PTI [7,8].

Pathogens have evolved a number of strategies to evade PTI such as the production of non-recognizable PAMPs [9], the scavenging of active PAMPs [10] or the interference with the PTI perception/signalling cascade. Inhibition of PTI can be achieved by bacterial toxins [11] or type III effector (T3E) proteins. In Gram-negative bacteria, T3E proteins are secreted and translocated directly into the host cell by the type III secretion (T3S) system [12]. Phytopathogenic bacteria of the genus *Ralstonia* , *Erwinia* , *Pseudomonas* and *Xanthomonas* express Hrp (Hypersensitive response and pathogenicity) T3S systems which are essential virulence determinants and secrete ca. 20-70 effectors inside the plant cell [13–15]. To date, most T3E for which molecular functions have been elucidated target PTI components resulting in effector-triggered susceptibility (ETS) [1]. For instance, the *Pseudomonas syringae* cysteine protease AvrPphB cleaves the RLCK (Receptor-like cytoplasmic kinases) PBS1 and several PBL (PBS1-like such as BIK1) proteins and inhibits FLS2-dependent PTI [7,16,17]. RLCK are kinases of RLK lacking extracellular and transmembrane domains compared to RLK. RLCK are often located at the plasma membrane due to palmitoylation/myristoylation or association with membrane proteins [18,19]. Two other *P. syringae* effectors AvrB and AvrRpm1 enhance phosphorylation of RIN4 (RPM1-interacting), a negative regulator of PTI [20–22]. RIN4 promotes stomatal opening *via* its interaction with two plasma membrane H^+^-ATPases [23]. Targeting RIN4 allows *P. syringae* to enhance plant stomatal opening, facilitating its entry inside the leaf.

Though important for ETS, T3E can also betray pathogens since their specific recognition by plant resistance (R) proteins causes effector-triggered immunity (ETI) [1]. ETI generally results in a hypersensitive response (HR) which is rapid and localized cell death at the infection site which limits pathogen growth and spread. R proteins essentially encode nucleotide-binding-site leucine-rich-repeat (NBS-LRR) proteins which usually recognize T3E indirectly by the modifications that the T3E induce on host proteins. For instance, the RPS5 R protein perceives the cleavage of PBS1 by AvrPphB [16]. Similarly, the RPM1 monitors the AvrB/AvrRpm1-dependent phosphorylation of RIN4 [20,22]. Conserved signalling modules downstream of R proteins usually require signalling hubs such as EDS1 (enhanced disease susceptibility) and PAD4 (phytoalexin-defficient) or NDR1 (non-race-specific disease resistance) [24]. While most results have been obtained on the mesophyll-colonizing *P. syringae*, relatively little is known about the mechanisms of plant immunity towards vascular pathogens.

The Gram-negative bacterium *Xanthomonas campestris* pv. *campestris* (Xcc) is a vascular pathogen and the causal agent of black rot disease in Brassicaceae [25]. *Xcc* can infect economically important crops like cabbage, mustard, radish, turnip, as well as the model plant *Arabidopsis thaliana*. *Xcc* colonizes and multiplies inside the xylem vessels after its entry *via* the hydathodes or wounds. The *Xcc* T3S system and the ca. 20-30 T3E called *Xanthomonas* outer proteins (Xop) are essential for bacterial pathogenicity [25,26]. XopAC (also called AvrAC) is a *Xanthomonas campestris*-specific type III effector. *xopAC* confers avirulence to *Xcc* only when bacteria are inoculated into the vascular system of the Columbia-0 (Col-0) ecotype of *A. thaliana* by piercing the main leaf vein, but not by mesophyll infiltration [27–29]. *xopAC* encodes a protein with an N-terminal LRR domain and a C-terminal fic (filamentation-induced by cAMP, consensus HPFxxG/ANGR) domain [28]. While the XopAC N terminus is sufficient to interact with the plant RLCK RIPK (RPM1-induced protein kinase) and BIK1, the XopAC fic domain uridylylates (transfer of uridine 5'-monophosphate; UMP) and inhibits the kinases [30]. BIK1 and RIPK are positive and negative regulators of PTI, respectively [7,17,31]. Importantly, *xopAC* deletion mutants in *Xcc* 8004 show reduced growth when infiltrated in leaves of cabbages and *Arabidopsis* ecotype Col-0 [30]. Because the fitness gain conferred by *xopAC* is BIK1-dependent in *Arabidopsis* , BIK1 inhibition seems to prevail over RIPK inhibition *in planta* [30]. In addition, RIPK is a positive regulator of the AvrB/RPM1-dependent ETI in *Arabidopsis* and mediates AvrB-induced RIN4 phosphorylation [31]. XopAC-mediated inhibition of RIPK by uridylylation [30] limits RIN4 phosphorylation monitored by RPM1 [31] and could explain the observed suppression of the AvrB/RPM1-dependent ETI in *Arabidopsis* [30]. To date, it remains unclear whether XopAC interacts with few or many members of the RLCK family. The plant genes required for resistance against *Xcc* and *xopAC*-mediated ETI are also as yet unknown.

In this study, we show that *xopAC* can confer avirulence to vascular and non-vascular bacterial pathogens. Several RLCK interacting with XopAC were identified by yeast two-hybrid assay. At least two of these RLCK are required to mount full resistance to *Xcc* and are distinct from the virulence-promoting RLCK inhibited by *xopAC* during a compatible interaction.

## Results

### The LRR and fic domains of XopAC are required for *Xcc* avirulence in *Arabidopsis* ecotype Col-0

**Figure 1:**
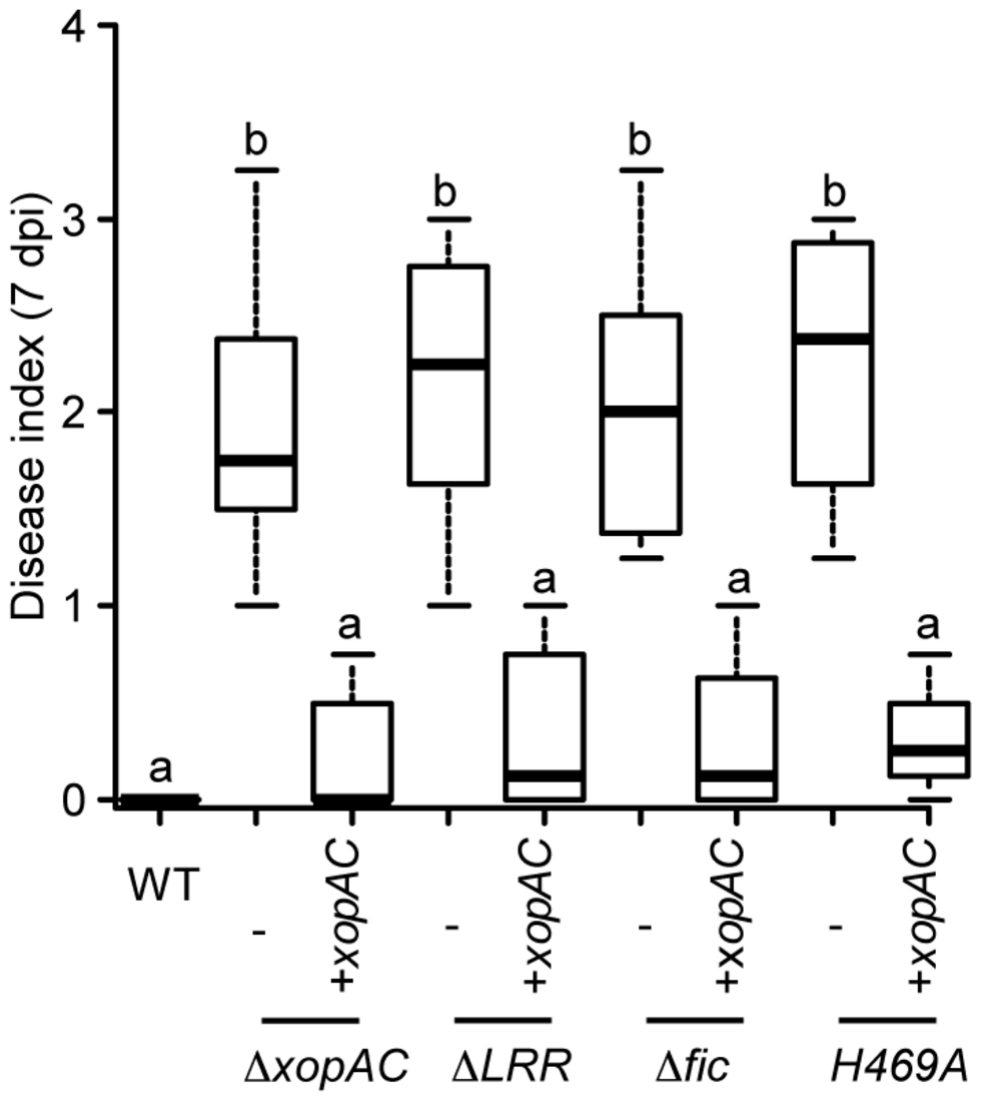
The LRR and fic domains of XopAC are required for XopAC-triggered immunity in *Arabidopsis* ecotype Col-0. A boxplot representation of pathogenicity of wild-type *Xcc* strain 8004 and *xopAC* mutants (∆*xopAC*, ∆*LRR*, ∆*fic*, *xopAC-H469A*) complemented or not with pCZ917-xopAC_A_ (+*xopAC*) is shown: middle bar = median; box limit = upper and lower quartile; extremes = Min and Max values. Bacteria were inoculated by piercing the leaf central vein and infection symptoms were scored 7 days post-inoculation. Disease index indicates: 0-1 no symptoms; 1-2 weak chlorosis, 2-3 strong chlorosis; 3-4 necrosis. N=3. Each time, at least 4 plants were inoculated on at least 3 leaves. Statistical groups were determined using a Tukey HSD test (*P*<0.001) and are indicated by a letter.

*xopAC* was previously reported to be essential for *Xcc* 8004 avirulence on Col-0 [28]. In order to determine which domains might be critical for the XopAC avirulence function on *Arabidopsis* ecotype Col-0, chromosomal deletions of the sequences coding for the LRR or fic domains were engineered in *Xcc* strain 8004. In parallel, the H469A mutation abolishing XopAC uridylylation activity [30] was also introduced in *xopAC*. Importantly, those mutations do not modify the XopAC N terminus so their secretion-translocation by the T3S system should not be affected [28]. The wild-type strain and the *xopAC* mutant derivatives were inoculated by piercing in the central vein of Col-0 (resistant to *Xcc* 8004) and Kas (susceptible to *Xcc* 8004) leaves. Seven days post-inoculation, 8004∆*xopAC*, 8004*xopAC*∆LRR, 8004*xopAC-*H469A and 8004*xopAC*∆fic were able to cause disease on Col-0 in contrast to the wild-type strain (Figure 1). These mutations were complemented by *xopAC*. On Kas, all strains were fully virulent (Supporting Figure S1). In order to test the expression level and the stability of these *xopAC* mutant alleles, chromosomal versions of the hrpG* (E44) [32] gain of function mutation were introduced in each strain. HrpG positively regulates a large regulon which includes the T3S system and most of its substrates in *X. campestris* pv. *vesicatoria* (Xcv) [33]. The HrpG* mutation allows the constitutive expression in rich medium of the T3S system and its substrates such as XopAC in *Xcc* (LDN, unpublished results). Immunoblot analysis using affinity purified anti-XopAC anti-serum demonstrated that these XopAC mutant proteins accumulated to detectable levels in total cell extracts of 8004::hrpG* (8004*, Supporting Figure S2A). These results indicate that the LRR domain, the fic domain and the uridylylation activity of XopAC are required for XopAC avirulence function on Col-0.

### *xopAC* Confers fic-dependent Avirulence to the Vascular Pathogen *Ralstonia solanacearum* in *Arabidopsis* Ecotype Col-0

**Figure 2:**
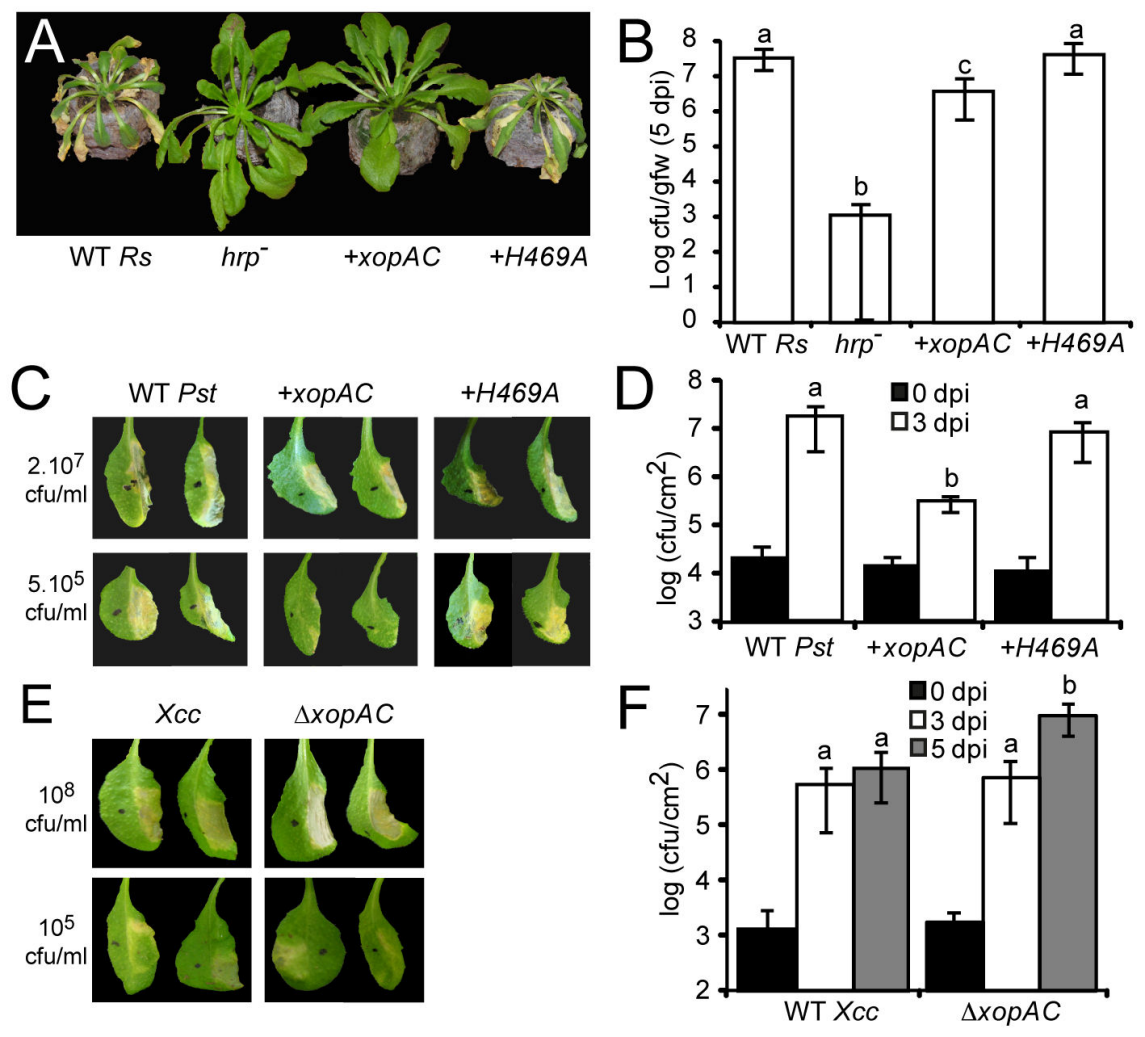
*xopAC* can confer avirulence to *Pst* strain DC3000 and *Rs* strain GMI100 on *Arabidopsis* ecotype Col-0. Four-week-old Col-0 plants were inoculated. (A, B) Wild-type and *hrcV* (*hrp* ^-^) mutant of *Rs* GMI1000 or strain derivatives carrying *xopAC* (+*xopAC*) or *xopAC-H469A* (+*H469A*) were inoculated by root dipping. (A) Pictures were taken at 11 days post-inoculation. (B) Bacterial populations in the aerial parts of the plants were determined at 5 dpi and expressed as log of colony-forming units per gram of fresh weight (cfu/gfw). For each strain, three samples of three plants each were analysed. Two independent experiments were performed. Statistical groups were determined using a Wilcoxon test (*P*<0.003) and indicated by different letters. (C, D) Leaves were infiltrated with wild-type *Pst* DC3000 or derivatives carrying pEDV6-xopAC (+*xopAC*) or pEDV6-xopAC-H469A (+*H469A*). (C) Bacterial suspensions of *Pst* at 2x10^7^ cfu/ml or 5x10^5^ cfu/ml were used and pictures were taken 3 days post-inoculation. (D) Bacterial suspensions at 5x10^5^ cfu/ml were infiltrated in leaves. *In planta* bacterial populations in the inoculated areas were determined 0 and 3 days post-inoculation and expressed as log (cfu/cm^2^). Standard deviations were calculated on two independent experiments with three samples of two leaf discs from different plants for each strain. Statistical groups were determined using a Wilcoxon test (*P*<0.012) and indicated by different letters. (E, F) Leaves were inoculated by hand infiltration with wild-type *Xcc* strain 8004 and 8004∆*xopAC*. (E) Bacterial suspensions of *Xcc* at 10^8^ cfu/ml or 10^5^ cfu/ml were used and pictures were taken 4 days post-inoculation. (F) *Xcc* strains were infiltrated at a bacterial density of 10^5^ cfu/ml. *In planta* bacterial populations in the inoculated areas were determined 0, 3 and 5 days post-inoculation and expressed as log (cfu/cm^2^). One representative experiment out of three is shown. Standard deviations were calculated on at least 4 biological samples. For each experiment, three samples of two leaf discs from different plants were collected for each strain. Statistical groups were determined using a Wilcoxon test (*P*<0.021) and indicated by different letters.

In order to determine whether *xopAC* can confer avirulence to other vascular pathogens, XopAC and its mutant derivatives were expressed in *Ralstonia solanacearum* (*Rs*) strain GMI1000, the causal agent of bacterial wilt in a wide range of plants. *xopAC* was expressed under the control of the promoter of the GALA7 effector [34]. *Xcc* and *Rs* T3S systems are closely related so proteins from one genus can be secreted by the T3S system of the other [35,36]. *Rs* strains expressing a wild-type or mutated *xopAC* gene were inoculated on wounded Col-0 roots. Wilting symptoms were then monitored (Figure 2A and B). While the WT strain wilted all plants by 11 days post-inoculation (dpi), the *hrcV* mutant and the strain expressing wild-type *xopAC* did not cause any visible wilting symptoms (Figure 2A). This *xopAC*-mediated avirulence was dependent on the H469 residue since the *Rs* strain expressing *xopAC*-H469A was fully virulent (Figure 2A). Finally, the *xopAC*-mediated avirulence of *Rs* was correlated with a significant decrease in bacterial growth (20-fold) but not as strong as the decrease observed with the *hrcV* mutant (Figure 2B). In conclusion, *xopAC*-mediated vascular ETI is fully efficient against another vascular bacterial pathogen and recruits the same functional domains as for *Xcc* 8004 avirulence on Col-0.

### *xopAC* confers fic-dependent avirulence to the mesophyll pathogen *P. syringae* on *Arabidopsis* ecotype Col-0

To learn whether *xopAC-*triggered immunity can also be observed in the mesophyllic tissues, *xopAC* was introduced in the mesophyll-colonizing pathogen *P. syringae* pv. *tomato* (*Pst*) strain DC3000. The pEDV6 vector was used to express fusion of the N-terminus of the *Pst* effector AvrRPS4 with XopAC to ensure its efficient delivery by the *Pst* T3S system [37]. Three days after infiltration of Col-0 leaves, *Pst* expressing the *xopAC* fusion caused reduced chlorotic symptoms compared to WT only when low inocula were employed (5x10^5^ colony-forming units (cfu)/ml; Figure 2C). Furthermore, this phenotype was dependent on H469 similarly to *Xcc* 8004 avirulence on Col-0. Importantly, the reduced chlorosis was correlated with reduced growth of the *Pst* strain expressing WT *xopAC* compared to a WT *Pst* strain or a *Pst* expressing *xopAC*-*H469A* (Figure 2D). XopAC and XopAC-H469A fusion proteins accumulated to comparable levels in total bacterial extracts (Supporting Figure S2C). These results demonstrate that *xopAC* can function as a bona fide avirulence protein in the mesophyll against a mesophyll-colonizing pathogen and that *xopAC*-induced ETI can be effective both in vascular and mesophyllic tissues.

### *xopAC* does not confer avirulence to *Xcc* when infiltrated in *Arabidopsis* ecotype Col-0

These results contrast with previous studies performed with *Xcc* [28]: *xopAC*-mediated avirulence in the mesophyll was not observed when high bacterial titres (10^8^ cfu/ml) were used for infiltration of Col-0 [28]. . Thus, WT strain 8004 and the ∆*xopAC* mutant were infiltrated in Col-0 and Kas plants at 10^8^ and 10^5^ cfu/ml. No differences of symptoms caused by the two strains were observed (Figure 2E, Supporting Figure S3A). Yet, growth of the ∆*xopAC* mutant was significantly increased at 5dpi compared to the WT strain when infiltrated at 10^5^ cfu/ml in Col-0 (Figure 2F, Supporting Figure S3B). Thus, *xopAC* is unable to confer macroscopic avirulence to *Xcc* in the mesophyll whatever the bacterial inoculum used, in contrast to our previous observations with *Pst*. Yet, *Xcc* multiplication in the mesophyll is reduced at 5 dpi by *xopAC* indicating that *Xcc* faces plant *xopAC*-triggered ETI in both the vasculature and the mesophyll during infection.

### XopAC localization at the plant plasma membrane is LRR-dependent

**Figure 3:**
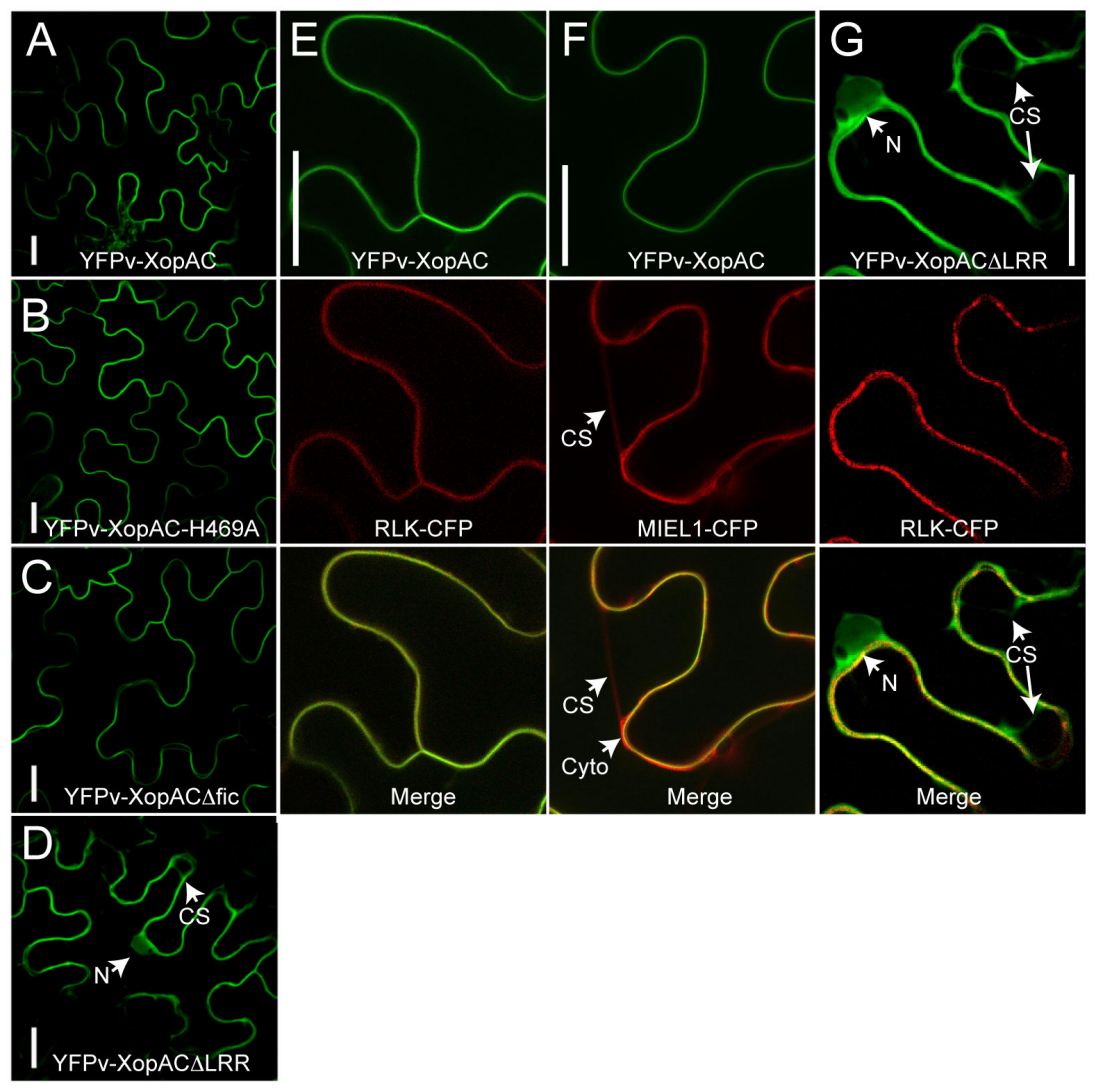
The LRR domain is required to target XopAC to the plasma membrane of *N* *. benthamiana* epidermal cells. YFPv-XopAC (A, E and F) and mutant variants (B, YFPv-XopAC-H469A; C, YFPv-XopAC∆fic and D, G, YFPv-XopAC∆LRR) were expressed using *Agrobacterium* -mediated transient transformation and imaged in epidermal cells by confocal laser microscopy 48 hours after inoculation. (A) The plasma membrane localized RLK-CFP fusion (At4g23740) and the nucleo-scytoplasmic marker MIEL1 (At5g18650) were co-expressed with YFPv-XopAC (E, F) or YFPv-XopAC∆LRR (G) and used as controls. The merged pictures are shown (E, F and G). Scale bars = 25 µm. White arrowheads indicate nuclei (N), cytosol (Cyto) and cytoplasmic strands (CS).

XopAC subcellular localization was determined by *Agrobacterium* -mediated expression of Yellow Fluorescent Protein venus (YFPv) fusions to XopAC variants (XopAC-H469A, XopAC∆LRR and XopAC∆fic) in *Nicotiana* *benthamiana* leaf epidermis cells. Accumulation and stability of the different YFPv-XopAC proteins was verified by Immunoblot analysis (Supporting Figure S2B). Interestingly, YFPv fusions to XopAC, XopAC-H469A and XopAC∆fic were localized at the plasma membrane while XopAC∆LRR was observed in nuclei, cytosol and cytoplasmic strands (Figure 3). XopAC plasma membrane localization was confirmed by co-localization with the RLK (At4g23740)-cyan-fluorescent protein (CFP) fusion known to be addressed to the plasma membrane (Figure 3E) [31]. In addition, YFPv-XopAC did not co-localize with the nucleocytoplasmic marker MIEL1-CFP (Figure 3F) [38]. Since no membrane-targeting signal or transmembrane domain was predicted in XopAC, its subcellular localization might be the result of an LRR-mediated interaction to a plasma membrane-associated protein.

### XopAC interacts with a subfamily of the *Arabidopsis* receptor-like cytosolic kinases (RLCK) in yeast two-hybrid assay

**Figure 4:**
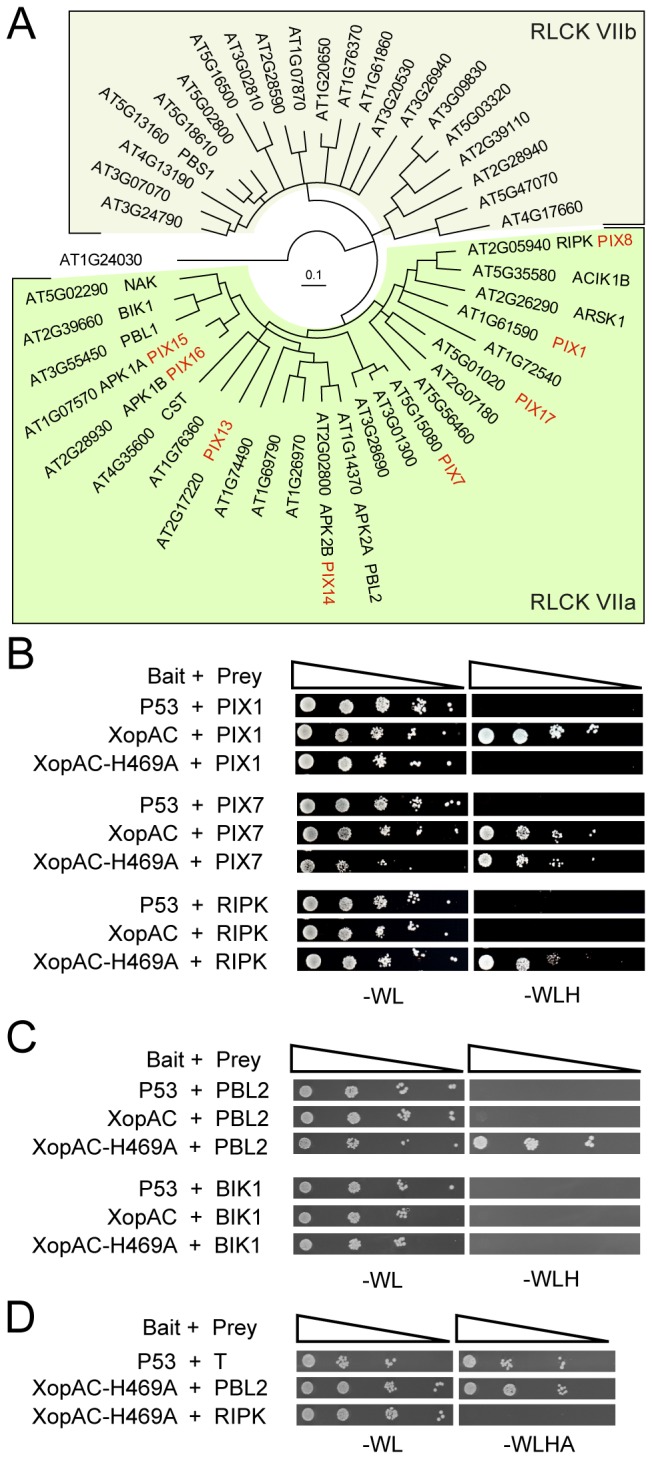
XopAC interacts with several members of the RLCK VIIa subfamily. (A) Neighbour-joining consensus tree of the 45 full-length *Arabidopsis* RLCK proteins aligned using Geneious alignment with default settings. AT1G24030 protein kinase was used to root the tree. Shaded areas define the two subfamilies VIIa and VIIb of RLK. PIX-RLCK proteins identified in the yeast two-hybrid screen with XopAC-H469A are indicated in red. Published protein names are indicated when available. (B, C, D) Yeast two-hybrid interaction tests between XopAC or its mutant allele H469A as bait and full-length PIX1, PIX7, PIX8-RIPK, PBL2 or BIK1 as prey. P53 was used as specificity control for the prey. Ten-fold serial dilutions of yeast transformants were spotted from left to right on minimal medium (-WL) and minimal medium without histidine (-WLH) or histidine and adenine (-WLHA) which were used to visualize prey/bait interaction. Pictures were taken 4 days after spotting.

In order to try and identify XopAC interactors, a LexA-based yeast two-hybrid screen [39] was performed against a normalized Col-0 cDNA library (*Arabidopsis thaliana* universal, 5.6 million clones, Dualsystems Biotech). Because the H348A mutation (equivalent of the H469A in XopAC) in the fic domain of VopS T3E from *Vibrio parahaemolyticus* was shown to stabilize the interaction with its substrate in pull-down assays [40], full-length XopAC-H469A was used as bait. From 2 million primary transformants, 68 bait-dependent interactors were isolated and corresponded to 50 putative interactors of XopAC (PIX) genes in the *Arabidopsis* genome. Remarkably, eight PIX genes (PIX 1, 7, 8, 13, 14, 15, 16 and 17) represented by eleven independent cDNA fragments (Table 1) encode RLCK belonging to the subfamily VII of the RLK [18]. To date, kinase activity was only described for two of these PIX-RLCK proteins (PIX8 and 15) [31,41]. RIPK/ACIK1A/PIX8 is the only interactor with a reported biological function in plant innate immunity [31] and a known interactor/substrate of XopAC [30]. More than 600 RLK have been identified in the Col-0 genome [18]. Although the functions of RLK are mostly unknown, RLK are proposed to be involved in nearly every aspect of plant life including growth, development and immunity [42]. Based on protein similarities, we have further subdivided the RLCK subfamily VII into VIIa and VIIb. Interestingly, all PIX-RLCK proteins belonged to subfamily VIIa (Figure 4A) in which the two known substrates of XopAC BIK1 ( *Botrytis* -induced kinase) [43] and RIPK can be found [30] as well as other players of plant innate immunity such as ACIK1B (Avr9/Cf-9 induced kinase) [44] or PBL1 (PBS1-like) [7]. Overlap between the different partial cDNA fragments identified in this yeast two-hybrid screen delimits a highly conserved minimal interaction domain of 45-46 residues (Supporting Figure S4). This region encompasses the kinase catalytic domain (domain VI) of the RLCK and is situated directly upstream of the uridylylation site of RIPK and BIK1 [30].

**Table 1:** List and properties of RLCK proteins identified by yeast two-hybrid assay as putative interactors with XopAC-H469A.

**PIX**	**Names**	**Gene**	**N° of cDNA**	**Protein domain**
PIX1	PBL15	AT1G61590	2	83-329 and 83-326
PIX7	-	AT5G15080	2	192-347 and 192-337
PIX8	RIPK/ACIK1A/PBL14	AT2G05940	2	85-240 and 81-240
PIX13	-	AT2G17220	1	58-241
PIX14	APK2B/PBL3	AT2G02800	1	44-250
PIX15	APK1A/PBL9	AT1G07570	1	15-217
PIX16	APK1B/PBL10	AT2G28930	1	30-245
PIX17	PBL8	AT5G01020	1	169-299

Interaction of full-length PIX8-RIPK with XopAC variants was tested in the yeast two-hybrid system. While RIPK/XopAC-H469A interaction was observed, no interaction with wild-type XopAC, XopAC∆LRR or XopAC∆fic was detected though proteins accumulated to similar levels by Immunoblot analysis (Figure 4B, Supporting Figure S5). The central kinase domain of RIPK (residues 87-367) interacted specifically with XopAC-H469A but not with wild-type XopAC (Supporting Figure S5A). Four other full-length RLCK PIX1, PIX7, BIK1 and PBL2 were tested for their interaction with XopAC. PIX1 interacted with wild-type XopAC but not with XopAC-H469A while PIX7 interacted with both the XopAC forms (Figure 4B and C). Finally, PBL2 was able to interact with XopAC-H469A but not wild-type XopAC while BIK1 did not interact to wild-type XopAC nor XopAC-H469A. Among all RLCK-XopAC interactions tested here, PBL2-XopAC-H469A interaction was the only one detectable on the selective medium lacking both histine and adenine (-WLHA) suggesting that PBL2-XopAC interaction is stronger than RIPK-XopAC. These results suggest that, besides RIPK and BIK1, XopAC might have the potential to interact with other members of the RLCK VIIa subfamily such as PBL2.

### PBL2 and RIPK are needed for *xopAC*-triggered immunity in Col-0

**Figure 5:**
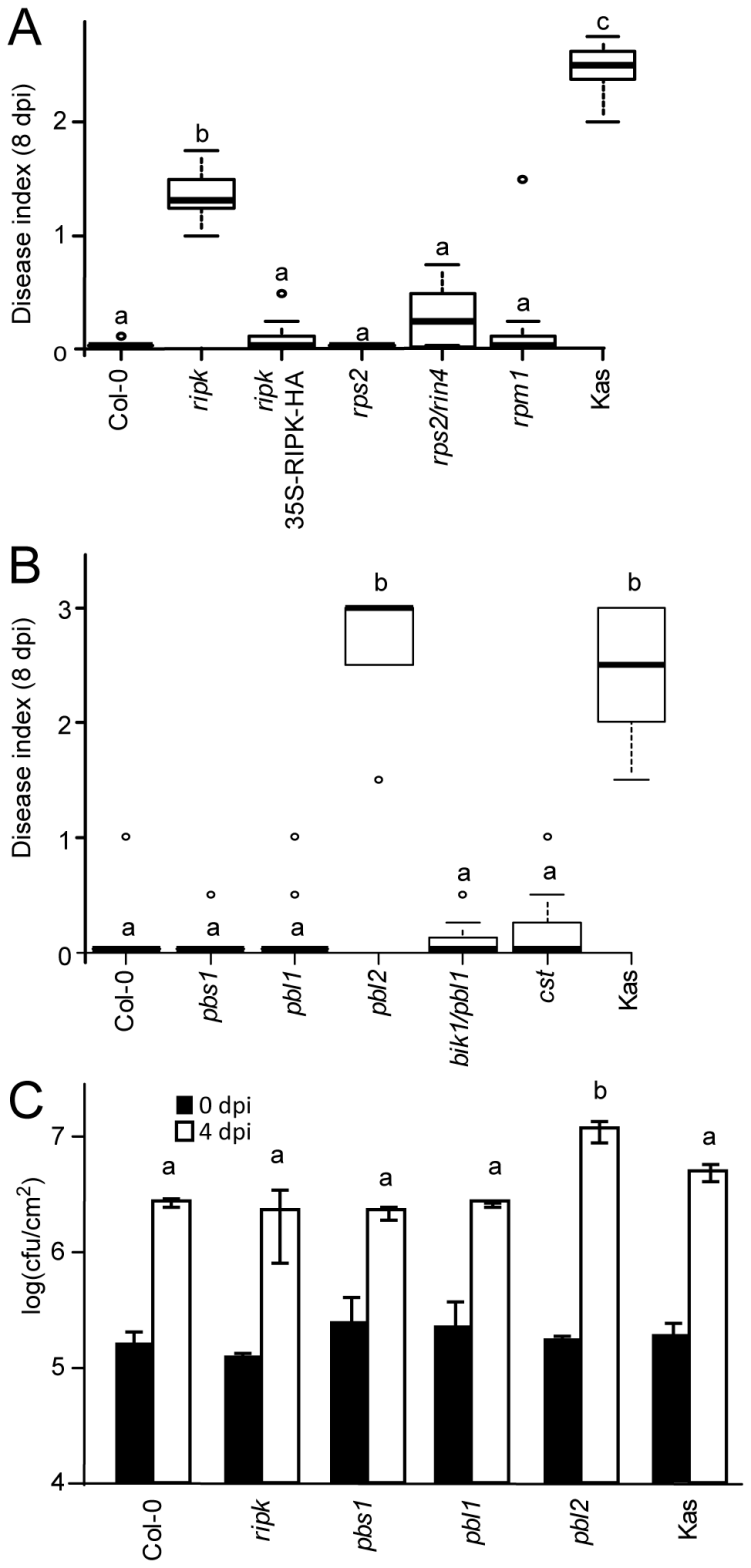
The RLCK genes *RIPK-PIX8* and *PBL2* are required for *xopAC*-mediated avirulence of *Xcc* strain 8004. (A,B) Boxplot representation of pathogenicity of strain 8004 on Col-0 mutants and transgenics inoculated by piercing the central vein of the leaves is shown: middle bar = median; box limit = upper and lower quartile; extremes = Min and Max values. Kas was used as a susceptible control. Mutants in genes coding for the RIPK-RIN4/RPM1 complex (A) and various RLCK (B) were tested. Disease indices were scored 8 days post-inoculation: 0-1 no symptoms; 1-2 weak chlorosis, 2-3 strong chlorosis; 3-4 necrosis. N=3. Each time, at least 4 plants were inoculated on at least 3 leaves. Statistical groups were determined using a Tukey HSD test (*P*<0.001) and are indicated by different letters. (C) A bacterial suspension (10^5^ cfu/ml) of *Xcc* strain 8004 was inoculated by piercing leaves of Col-0 mutants and transgenics. *In planta* bacterial populations in the inoculated areas were determined 0 and 4 days post-inoculation and expressed as log of colony-forming units per square cm (cfu/cm^2^). Standard deviations were calculated on two independent experiments. For each experiment, three samples of two leaf discs from different plants were collected for each strain. Statistical groups identified using a Wilcoxon test (*P*<0.05) are indicated by different letters.

To determine whether these RLCK might be important to mount resistance against *Xcc* strains expressing *xopAC*, several RLCK mutants (*ripk*, *pbs1*, *pbl1*, *pbl2*, *bik1/pbl1* and *cst*) were inoculated with *Xcc* strain 8004 by piercing. The *CAST AWAY* (*CST*) gene encodes an *RLCK* of subfamily VIIa which is important for organ abscission [19]. *cst* mutant was included as a negative control. Eight days post-inoculation, the *ripk* and *pbl2* mutants developed significant disease symptoms (Figure 5A and B). Yet, the *ripk* mutant was less susceptible than the ecotype Kas or the *pbl2* mutant. The *ripk* mutant was complemented by overexpression of RIPK-HA indicating that *RIPK* is genetically important for *xopAC*-triggered immunity. Interestingly, *RIPK* positively regulates *RPM1*-/*RIN4*-dependent *avrB*-triggered immunity [31]. Because XopAC and AvrB are both members of the fido family (fic, doc and AvrB) [45] and might share other signalling components, the *rpm1* and the *rps2*/*rin4* mutants were inoculated with strain 8004 by piercing (Figure 5A). The *rps2*/*rin4* mutant was used because a *rin4* mutant is not viable [20]. These two mutants were as resistant as the wild-type Col-0 plants. In agreement with these observations, *in planta* growth of strain 8004 was significantly higher in the *pbl2* mutant (Figure 5C). Bacterial populations in the *ripk*, *pbs1* and *pbl1* mutants and the wild-type plants were comparable. These *Arabidopsis* mutants showed wild-type susceptibility to the *Xcc* 8004∆*xopAC* mutant strain (Supporting Figure S6). 8004∆*xopAC* growth was not affected in the different *Arabidopsis* mutants tested (Supporting Figure S6). These results suggest that *PBL2*, and to a lesser extent *RIPK*, positively regulate *xopAC*-triggered immunity.

## Discussion

### XopAC can trigger ETI against both vascular and mesophyll-colonizing bacterial pathogens

Previous studies have shown that *xopAC* can restrict *Xcc* growth in the vasculature of Col-0 but not in Sf-2 ecotypes of *Arabidopsis* [27,28]. In this study, we provide evidence that *xopAC* is able to confer avirulence to other phytopathogenic bacteria. In particular, the vascular pathogen *Rs* expressing XopAC did not cause any wilting symptoms on Col-0 plants similar to a T3S mutant. Bacterial populations in plant shoots were also significantly reduced. This indicates that the immune responses triggered by XopAC can be efficient against other vascular pathogens and that the genetic dissection of this particular ETI could help to identify novel targets to manage/control other vascular pathogens such as *Rs*. XopAC also conferred avirulence to *Pst* DC3000 in Col-0 ecotype but not in Kas when infiltrated at low bacterial densities. This result indicates that *xopAC* has the potential to be recognized in the mesophyll and to trigger potent ETI in this tissue. Yet, such ETI was not observed macroscopically when *Xcc* was infiltrated even at low bacterial densities in leaf mesophyll (Figure 4E) [27,28]. Interestingly, an increased growth of the ∆*xopAC* mutant was reproducibly observed on Col-0. This observation suggests that *Xcc* encounters *xopAC*-triggered immunity not only in *Arabidopsis* vasculature, but also in the leaf mesophyll (Figure 4F). The number of inoculated bacteria (a few hundred by piercing versus millions by infiltration) might explain why a strong avirulence is observed by piercing but not by infiltration of *Xcc*. In conclusion, *xopAC* cannot be considered as a vasculature-specific avirulence determinant which indicates that its pathogenicity and/or avirulence targets should be found both in mesophyll and vascular tissues.

### XopAC avirulence functions likely depend on host target uridylylation

No rapid HR-like symptoms were observed when *Xcc* or *Pst* expressing *xopAC* were infiltrated into Col-0 leaf tissues. Furthermore, in *Pst*, *xopAC* avirulence was symptomless (Figure 2C). These observations suggests that *xopAC*-triggered immunity might not rely on rapid cell death and that it might differ mechanistically from other ETIs: Interestingly, resistance against bacterial pathogens can be achieved without plant cell death [46] and *xopAC*-triggered ETI might be too weak to induce HR as exemplified by *TAO1*-dependent recognition of *avrB* [47]. Importantly, the XopAC fic domain and its conserved H469 residue which are important for UMP transferase activity *in vitro* [30] are also essential for the *xopAC* avirulence function: XopAC uridylylation activity cannot be uncoupled from XopAC biological functions. Thus, *xopAC*-triggered immunity likely results from host target uridylylation.

### Plasma membrane-localized XopAC interacts with members of the VIIa RLCK subfamily

In our search for XopAC targets, we identified eight *Arabidopsis* RLCK proteins as the major class of XopAC-H469A interactors by yeast two-hybrid assay. The H469A mutation was used since the orthologous mutation in VopS was shown to stabilize the interaction with Rho-GTPases [40]. Yet, validation of the interactions with full length RLCK cDNAs showed that the interaction could be reconstituted with wild-type or mutant XopAC or both depending on the RLCK studied. The biological relevance of some of these interactions was recently demonstrated by the identification of *xopAC*-dependent uridylylation of three RLCK (BIK1, PBL1 and RIPK-PIX8) resulting in the inhibition of their kinase activity [30]. Several RLCK like BIK1 and to a lesser extent PBL1 and PBL2 are positive regulators of PTI downstream of EFR, FLS2 and CERK1 while RIPK is a negative regulator of PTI [7,31]. Several RLCK like RIPK, PBL2 or CST are localized at the plasma membrane and many possess predicted myristoylation or palmitoylation sequences in their N-terminal domain [7,19,31]. For instance, PBL2-YFP can be myristoylated *in vitro* and its plasma membrane localization in tobacco depends on this myristoylation site [48]. In *Arabidopsis* , proteomic studies of plasma membrane-enriched fractions identified PBL2 [49] and PBL2 can be co-immunoprecipitated with the plasma membrane receptor FLS2 [7]. Therefore, there is compelling evidence for PBL2 localization at the plasma membrane. Interestingly, XopAC also localizes at the plasma membrane in *N* *. benthamiana* leaves though lacking membrane-anchoring motifs. This association to the plasma membrane is dependent on the XopAC highly conserved LRR domain which is needed to interact with BIK1 and RIPK [30]. Thus, it is tempting to propose that XopAC subcellular localization is achieved by interaction of its N-terminal-LRR domains with membrane-associated RLCK. In our yeast-two hybrid screen, only cDNAs of RLCK subfamily VIIa were identified. Its substrates BIK1, PBL1 and RIPK also belong to that group. These results suggest that XopAC associate preferentially to RLCK subfamily VIIa though the ST or TT uridylylated residues are highly conserved throughout subfamily VII. *In planta* interaction studies as well as uridylylation tests will be needed to address this question. In the PTI context, BIK1 probably remains the most relevant XopAC target. Because RLCK are highly conserved, interaction with them does not have to be highly specific to a few biologically important RLCK: a global inhibition of all RLCK subfamily VIIa members including BIK1, PBL1, PBL2 and RIPK would be sufficient to achieve PTI suppression. Such inhibition of RLCK subfamily VIIa by XopAC is very reminiscent of the proteolytic cleavage of many RLCK subfamily VII members by AvrPphB [7]. This observation further defines the RLCK family as a prime pathogenicity target for bacterial pathogens to suppress PAMP-triggered immunity or other RLCK-dependent physiological responses.

### *xopAC*-dependent ETI in *Arabidopsis* depends on RIPK and PBL2

While *xopAC*-mediated suppression of PTI is rather well understood, the mechanisms required for *xopAC*-triggered immunity against *Xcc* are lacking. In this study, PBL2 was the strongest interactor of XopAC among all RLCK tested in yeast-two hybrid (Figure 4D). Interestingly, we identified the *PBL2 RLCK* gene as critical components of this ETI and RIPK to a lesser extent. ETI suppression in the *pbl2* mutant is unexpected since this kinase was so far only known as a positive PTI regulator partially needed to respond to flg22 and efl18 but not chitin [7]. Yet, *pbl2* was fully susceptible to *Xcc* strain 8004 expressing *xopAC* both in terms of disease symptoms and bacterial growth. Importantly, *pbs1*, *pbl1, bik1/pbl1* or *cst* mutants were fully resistant suggesting that *PBL2* is specifically recruited to mount this ETI. The *ripk* mutant showed an intermediate susceptibility to *Xcc* strain 8004 for disease symptoms, but sustained wild-type bacterial growth. RIPK is needed for the *RPM1*-specified ETI, interacts with AvrB and RIN4 and can phosphorylate both AvrB and RIN4 [31]. Phosphorylation of RIN4 then activates RPM1 and the subsequent ETI response. In a compatible interaction between *Xcc* and its host, XopAC can uridylylate RIPK and consequently suppress the *RPM1*-specified ETI [30]. The partial susceptibility of the *ripk* mutant could be explained in several ways: Functional redundancy of *RIPK* with one of its close homologues (such as ACIK1B/PBL13) is an obvious option. Also, RIPK acts as a negative regulator of PTI so its inhibition might mask ETI breakdown. Whether *PBL2*- and *RIPK*-dependent ETI act independently or additively is not known. Interestingly, resistance to *Xcc* did not genetically require *RIN4* nor *RPM1*. On the one hand, RIPK could signal through *RIN4/RPM1*-independent signalling cascades yet to be identified. On the other hand, genetic redundancy in the RIN4 and RIN4-like proteins might blur our analyses [50]. Further experimental evidence will be needed to establish the recruitment of the *RIN4/RPM1* signalling module in *xopAC*-triggered immunity. To conclude, our results are very reminiscent of PBS1 being a decoy guarded by RPS5 to detect AvrPphB-dependent degradation of other RLCK important for PTI [7,51]. During the *xopAC*-mediated ETI against *Xcc* in *Arabidopsis* , PBL2 and/or RIPK could well be decoys detecting uridylylation and inhibition of RLCK important for plant innate immunity such as BIK1.

## Materials and Methods

### Bacterial strains, plasmids and growth conditions

Strains and plasmids used in this study are listed in Supporting Table S1. *Xcc*, *A* *. tumefasciens* , *Rs* and *Pst* strains were grown at 28° C in MOKA [52], in yeast extract-beef medium (YEB), complete medium BG [53] and King’s B medium [54], respectively. *Escherichia coli* cells were grown on Luria-Bertani medium at 37° C. For solid media, agar was added at a final concentration of 1.5% (w/v). Antibiotics were used at the following concentrations: for all bacteria, 50 µg/ml kanamycin, 50 µg/ml rifampicin, 50 µg/ml ampicillin; for *Xcc*, 5 µg/ml tetracycline; for *A. tumefaciens*, 15 µg/ml gentamycin, 100 µg/ml spectinomycin and 5 µg/ml tetracycline; for *Pst*, 50 µg/ml gentamycin; for *Rs*, 40 µg/ml spectinomycin, 5 µg/ml gentamycin; for *E. coli*, 40 µg/ml spectinomycin and 10 µg/ml tetracycline.

### Plant material and growth conditions

Wild-type Col-0 and Kas ecotypes were used as well as mutants in the Col-0 genetic background: *ripk* (GT22343 backcrossed into Col-0) and *ripk* 35S–RIPK*-HA* (line 16-5) [31], *rps2-101C* [55], *rps2-101C*/*rin4* [56], *rpm1-3* [57], *pbs1-1* [58], *pbl1* (SAIL_1236_D07), *pbl2* (SALK_149140) [7] and *cst-1* [19]. *Arabidopsis* plants were grown on Jiffy pots in a growth chamber at 22° C, with a 9-h light period and a light intensity of 192 µmol.m^-2^.s^-1^. After 2 days at 4° C, plates were transferred to a growth chamber at 20° C with 16-h light period. *N* *. benthamiana* plants were grown in temperate greenhouses.

### Pathogenicity tests

*Xcc* pathogenicity was assayed on the *A. thaliana* accessions Columbia (Col-0) and Kashmir (Kas) by piercing inoculation of bacterial suspension at 10^8^ cfu/ml as described [27]. Each strain was tested on 4 plants with 4 leaves per plant. *Xcc* pathogenicity was also assayed on *A. thaliana* by infiltration of bacterial suspensions at 10^5^ and 10^8^ cfu/ml as described [27].

*Pst* pathogenicity was assayed on *A. thaliana*, by infiltration of bacterial suspensions at 2x10^7^ cfu/ml. Each strain was tested on 6 plants with 4 leaves per plant. After inoculation, plants were kept at almost 100% relative humidity.

*Rs* pathogenicity was assayed on *A. thaliana* Col-0 by root inoculations essentially as described [59]: Approximately 2 cm was cut from the bottom of the Jiffy pot and the exposed roots of the plants were immersed for 15 min in a bacterial suspension containing 10^7^ bacteria/ml. The plants were then transferred to a growth chamber at 27° C (16 h light): 26° C (8 h dark) until symptom appearance. Disease development was scored daily, using a macroscopic scale describing the observed wilting: 1 for 25% of the leaves wilted; 2 for 50%; 3 for 75% and 4 for complete wilting. The plants were grown at 25° C.

At least three independent repetitions were performed for all pathogenicity tests. Statistical analysis was performed using a fixed effect model and a Tukey HSD test with correction for multiple testing. All analyses were performed using R software version 2.14.1 (www.r-project.org).

### Determination of *in planta* bacterial populations

For *Xcc* and *Pst*, plant leaves were infiltrated with a bacterial suspension of 10^5^ cfu/ml and 5x10^5^ cfu/ml, respectively. Two leaf discs from distinct infiltrated leaves of different inoculated plants were sampled using a cork borer (area = 0.33 cm^2^) at 0, 3, 4 or 5 days after inoculation and were homogenized in 200 µl sterile water. Serial dilutions of the homogenates were performed and 5 µl drops were spotted in triplicate for each dilution on plates supplemented with appropriate antibiotics. The plates were incubated at 28° C for 48 h and colonies were counted in spots containing 1 to 30 colonies [28].

For *R. solanacearum*, the *in planta* bacterial populations were determined as described [59] with the following modifications: plants were root-inoculated with a bacterial suspension containing 10^7^ cfu/ml. The aerial parts of three whole inoculated plants were weighed, surface sterilized three times in 250 ml of 70% ethanol, rinsed twice in sterile water and ground in sterile water. Bacterial densities in leaves were determined by plating serial dilutions on complete BG medium.

At least two independent experiments were performed.

### Construction of *xopAC* and *RLCK* ENTRY clones

All oligonucleotides mentioned in this study are described in Supporting Table S2. XopAC open reading frame (with stop codon) was amplified from genomic DNA of wild-type *Xcc* 8004 with Phusion DNA polymerase (Finnzymes, Vantaa, Finland) using MC12/13 primers. xopAC∆LRR and xopAC∆fic amplicons were produced by fusion-PCR between amplicons MC4/12+MC5/13 and MC8/12+MC9/13, respectively. *att*B1 and *att*B2 sites were added with a third PCR using MC18/MC19 oligonucleotides. Wild-type and *xopAC* deletion mutant amplicons were recombined by BP reaction into pDONR207 (Invitrogen) giving pENTRY-xopAC, pENTRY-xopAC∆LRR and pENTRY-xopAC∆fic. pENTRY-xopAC-H469A was produced by site-directed mutagenesis of pENTRY-xopAC with primers LN433/434 using the QuikChange mutagenesis kit (Stratagene). *PIX1*, *PIX7, PIX8* (full length and amino acids 87-367), BIK1 and PBL2 ORFs (with stop codon) were amplified from cDNA of Col-0 seedlings grown *in vitro* using primers EG45/46, EG58/59, EG62/63, EG167/168, LN714/715 and LN716-717, respectively and recombined by BP into pDNOR207 giving pENTRY-PIX1, pENTRY-PIX7, pENTRY-PIX8 and pENTRY-PIX8_87-367_, pENTRY-BIK1 and pENTRY PBL2.

### Construction of *xopAC* and hrpG* mutant derivatives in *Xcc* 8004

Deletion mutants or gene replacements in *Xcc* 8004 were obtained using the SacB system with p∆13 suicide vector (L. Noël, unpublished), a pK18 derivative [60] designed for BsaI-based Goldengate cloning [61]. Plasmids were introduced into E. coli by electroporation and into *Xcc* by triparental mating as described [62,63]. Deletion events were verified by PCR (details available upon request). Standard methods were used unless stated differently.

In order to introduce the HrpG E44K mutation in *Xcc* 8004 genome, XC_3077 gene fragment was amplified with LN251/252 from 8004 genomic DNA and cloned into p∆13 giving p∆13-hrpG. Site-directed mutagenesis was performed using LN202/203 giving p∆13-hrpG* and introduced in wild-type strain 8004 and 8004∆*hrcV* (L. Noël, unpublished).

To engineer xopAC∆LRR genomic deletion, site-directed mutagenesis of one internal *Bsa*I restriction site in pENTRY-xopAC∆LRR was performed using EG3/4. The EG5/6 amplicon was cloned by Goldengate into p∆13 giving p∆13-xopAC∆LRR. For xopAC∆fic deletion, site-directed mutagenesis of one internal *Bsa*I restriction site in pENTRY-xopAC was performed using EG3/4. The EG7/MC8 and EG8/MC9 amplicons were assembled by Goldengate into p∆13 giving p∆13-xopAC∆fic. For H469 mutagenesis, amplicons LN436/9 and LN442/443 were cloned in p∆13 and subjected to site-directed mutagenesis with LN433/434, giving p∆13-xopAC-H469A.

### XopAC expression vectors for *Pst* and Rs

For expression and efficient typeIII-dependent translocation XopAC in *Pst*, *xopAC* and *xopAC-H469A* ENTRY clones were recombined by LR into pEDV6 [37] giving pEDV6-xopAC and pEDV6-xopAC-H469A, respectively. Plasmids were introduced in *Pst* by triparental mating. For expression in *Rs*, ENTRY clones of wild-type and mutant *xopAC* were recombined by LR into pNP269 (N. Peeters, unpublished) destination vector. Plasmids were introduced in *Rs* by natural transformation as described [64].

### Transient expression of YFPv-XopAC fusions in N benthamiana

N-terminal fusions of YFPv to wild-type and mutant XopAC were generated by LR recombination of the corresponding ENTRY vectors in pBin19-35S-YFPv-GW (S. Rivas, unpublished) giving pBin19-YFPv-xopAC, pBin19-YFPv-xopAC∆LRR, pBin19-YFPv-xopAC∆fic and pBin19-YFPv-xopAC-H469A. Plasmids were transformed in Chemo-competent C58C1 *Agrobacterium* cells. *Agrobacterium* -mediated transient expression in *N* *. benthamiana* leaves was performed as described [65]. Plants were grown in a growth chamber at 21° C, with a 16-h light period and 70% hygrometry.

### Fluorescence microscopy

YFPv fluorescence in *N* *. benthamiana* leaves was analysed with a confocal laser scanning microscope (TCS SP2-SE, Leica) using a x40 water immersion objective lens (numerical aperture 1.20; PL APO) or the x63 oil immersion objective lens (numerical aperture 1.40; PL APO). Fluorescence of YFPv and CFP was excited with the 514 nm and 458 nm rays of the argon laser and detected between 520 and 575 nm and 465 and 520 nm, respectively. Images were acquired in the sequential mode (20 Z planes per stack of images; 0.5 mm per Z plane) using Leica LCS software (version 2.61).

### Identification of XopAC interactors by yeast two-hybrid approach

A LexA-based yeast two-hybrid screen using the full-length XopAC-H469A (Amplicon LN608-609 cloned *Pst*I/*Xma*I in the bait vector pLexA-DIR) was carried out by Dualsystems Biotech AG, Zurich, Switzerland. The bait construct was transformed into the strain NMY32 using standard procedures [66]. Correct expression of the bait was verified by immunoblotting of cell extracts using a mouse monoclonal antibody directed against the LexA domain (Dualsystems Biotech, Switzerland). The absence of self-activation was verified by co-transformation of the bait together with a control prey and selection on minimal medium lacking the amino acids tryptophan, leucine and histidine (selective medium). For the yeast two-hybrid screen, the bait was co-transformed together with a normalized *Arabidopsis* universal cDNA library (Col-0; Dualsystems P02403) into NMY32. 2x10^6^ transformants were screened yielding 85 transformants that grew on selective medium. Positive transformants were tested for β-galactosidase activity using a PXG β-galactosidase assay (Dualsystems Biotech). 68 of the 85 initial positives showed β-galactosidase activity and were considered to be true positives. Library plasmids were isolated from positive clones. The identity of positive interactors was determined by sequencing and comparison to the *Arabidopsis* databases.

Pairwise interactions between XopAC and derivatives vs. PIX-RLCK were tested in the GAL4 yeast two-hybrid system (Clontech). To this end, pENTRY clones of wild-type XopAC and H469A mutant derivative were recombined by LR in pGBKT7-GW (Clontech, Gateway-compatible derivative, L. Deslandes) to generate pGBKT7-xopAC and pGBKT7-xopAC-H469A. pENTRY-PIX1, pENTRY-PIX7, pENTRY-PIX8, pENTRY-PIX8_87-367_, pENTRY-BIK1 and pENTRY-PBL2 were recombined by LR into a Gateway-compatible pGADT7 (Clontech, L. Deslandes). *Saccharomyces cerevisiae* strain AH109 were co-transformed with bait and prey vectors as described (Clontech). Auto-activation of the His auxotrophy reporter was tested on SD-WLH (Supporting Figure S4B). Interactions were tested on at least 3 different clones.

### XopAC antibody preparation

pENTRY-xopAC was recombined by LR in pDEST17 giving pDEST17-xopAC and transformed in *E. coli* BL21 (DE3) pLysS. A culture in exponential phase was treated with 2mM IPTG for 2 hrs and cells were harvested by centrifugation. Inclusion bodies of His_6_-tagged XopAC were solubilized in urea-containing buffer, loaded on TALON resin as recommended (Clontech) and eluted with 200mM imidazole. One mg of His-XopAC was used for the immunisation of two rabbits (GenCust, Dudelange, Luxemburg). Anti-serum was purified on nitrocellulose-immobilized His-XopAC and used at dilutions of 1/1000 (Plant extracts) or 1/5000 (other extracts) for XopAC detection by immunoblotting on plant and bacterial protein samples. A specific signal at the expected size was detected in 8004* strain grown in MOKA but not 8004*∆*xopAC* (Supporting Figure S2A).

### Preparation of protein extracts, SDS-PAGE and immunoblotting

Yeast protein extracts were prepared under denaturing conditions as described [67] with the following modifications: after washing in buffer A, the cells were frozen twice in liquid nitrogen. After the last centrifugation step, the cells are washed with 100% ethanol, centrifuged at 11000 g and resuspended in 1X Laemmli buffer (0.5 M Tris pH 6.8, SDS 10%, glycerol 20% and 0.2 M DTT). Total soluble protein extracts were prepared from *N* *. benthamiana* leaf tissues as described [68]. Bacterial protein extracts were prepared by resuspending bacterial pellets directly in 1X Laemmli buffer. Samples were separated by SDS-PAGE (8 or 10% polyacrylamide) and transferred to nitrocellulose. The following antibodies were used: rat anti-HA (1867423; Roche), rabbit anti-GFP (B. Lefebvre, unpublished), mouse anti-c-Myc (Santa Cruz). Secondary antibodies were purchased from Sigma (AP conjugates) and detected as described [68].

## Supporting Information

Figure S1 The LRR and fic domains of XopAC are not required for pathogenicity on *Arabidopsis* ecotype Kas.
(PDF)

Figure S2 Accumulation and stability of XopAC variants expressed in *Xcc* 8004*, *Pst* DC3000 and *N* *. benthamiana* .
(PDF)

Figure S3 Symptom development and *in planta* growth of *Xcc* strain 8004 infiltrated into leaves of *Arabidopsis* ecotype Kas are not affected by XopAC.
(PDF)

Figure S4 Protein alignment of the eight full-length PIX proteins belonging to the RLCK family identified by yeast two-hybrid assay as putative interactors with XopAC-H469A.
(PDF)

Figure S5 XopAC-H469A interacts with the PIX8 kinase domain in a yeast two-hybrid assay.
(PDF)

Figure S6 Pathogenicity and *in planta* growth of strain 8004∆*xopAC* on Col-0 mutants and transgenics inoculated by piercing the central leaf vein.
(PDF)

Table S1 Strains and plasmids used in this study.
(PDF)

Table S2 Oligonucleotides used in this study.
(PDF)

## References

[1] Jones JD, Dangl JL (2006) The plant immune system. Nature 444: 323-329. doi:10.1038/nature05286. PubMed: 17108957. 17108957 10.1038/nature05286

[2] Zipfel C, Felix G (2005) Plants and animals: a different taste for microbes? Curr Opin Plant Biol 8: 353-360. doi:10.1016/j.pbi.2005.05.004. PubMed: 15922649. 15922649 10.1016/j.pbi.2005.05.004

[3] Ronald PC, Beutler B (2010) Plant and animal sensors of conserved microbial signatures. Science 330: 1061-1064. doi:10.1126/science.1189468. PubMed: 21097929. 21097929 10.1126/science.1189468

[4] Gómez-Gómez L, Boller T (2000) FLS2: an LRR receptor-like kinase involved in the perception of the bacterial elicitor flagellin in Arabidopsis. Mol Cell 5: 1003-1011. doi:10.1016/S1097-2765(00)80265-8. PubMed: 10911994. 10911994 10.1016/s1097-2765(00)80265-8

[5] Zipfel C, Kunze G, Chinchilla D, Caniard A, Jones JD et al. (2006) Perception of the bacterial PAMP EF-Tu by the receptor EFR restricts *Agrobacterium*-mediated transformation. Cell 125: 749-760. doi:10.1016/j.cell.2006.03.037. PubMed: 16713565. 16713565 10.1016/j.cell.2006.03.037

[6] Danna CH, Millet YA, Koller T, Han SW, Bent AF et al. (2011) The Arabidopsis flagellin receptor FLS2 mediates the perception of *Xanthomonas* Ax21 secreted peptides. Proc Natl Acad Sci U S A 108: 9286-9291. doi:10.1073/pnas.1106366108. PubMed: 21576467. 21576467 10.1073/pnas.1106366108 PMC3107323

[7] Zhang J, Li W, Xiang T, Liu Z, Laluk K et al. (2010) Receptor-like cytoplasmic kinases integrate signaling from multiple plant immune receptors and are targeted by a *Pseudomonas syringae* effector. Cell Host Microbe 7: 290-301. doi:10.1016/j.chom.2010.03.007. PubMed: 20413097. 20413097 10.1016/j.chom.2010.03.007

[8] Chinchilla D, Zipfel C, Robatzek S, Kemmerling B, Nürnberger T et al. (2007) A flagellin-induced complex of the receptor FLS2 and BAK1 initiates plant defence. Nature 448: 497-500. doi:10.1038/nature05999. PubMed: 17625569. 17625569 10.1038/nature05999

[9] Sun W, Dunning FM, Pfund C, Weingarten R, Bent AF (2006) Within-species flagellin polymorphism in *Xanthomonas campestris* pv *campestris* and its impact on elicitation of Arabidopsis FLAGELLIN SENSING2-dependent defenses. Plant Cell 18: 764-779. doi:10.1105/tpc.105.037648. PubMed: 16461584. 16461584 10.1105/tpc.105.037648 PMC1383648

[10] de Jonge R, van Esse HP, Kombrink A, Shinya T, Desaki Y et al. (2010) Conserved fungal LysM effector Ecp6 prevents chitin-triggered immunity in plants. Science 329: 953-955. doi:10.1126/science.1190859. PubMed: 20724636. 20724636 10.1126/science.1190859

[11] Melotto M, Underwood W, Koczan J, Nomura K, He SY (2006) Plant stomata function in innate immunity against bacterial invasion. Cell 126: 969-980. doi:10.1016/j.cell.2006.06.054. PubMed: 16959575. 16959575 10.1016/j.cell.2006.06.054

[12] Galán JE, Wolf-Watz H (2006) Protein delivery into eukaryotic cells by type III secretion machines. Nature 444: 567-573. doi:10.1038/nature05272. PubMed: 17136086. 17136086 10.1038/nature05272

[13] Lindeberg M, Cunnac S, Collmer A (2012) *Pseudomonas syringae* type III effector repertoires: last words in endless arguments. Trends Microbiol 20: 199-208. doi:10.1016/j.tim.2012.01.003. PubMed: 22341410. 22341410 10.1016/j.tim.2012.01.003

[14] White FF, Potnis N, Jones JB, Koebnik R (2009) The type III effectors of *Xanthomonas* . Mol Plant Pathol 10: 749-766. doi:10.1111/j.1364-3703.2009.00590.x. PubMed: 19849782. 19849782 10.1111/j.1364-3703.2009.00590.x PMC6640274

[15] Poueymiro M, Genin S (2009) Secreted proteins from *Ralstonia solanacearum*: a hundred tricks to kill a plant. Curr Opin Microbiol 12: 44-52. doi:10.1016/j.mib.2008.11.008. PubMed: 19144559. 19144559 10.1016/j.mib.2008.11.008

[16] Shao F, Golstein C, Ade J, Stoutemyer M, Dixon JE et al. (2003) Cleavage of Arabidopsis PBS1 by a bacterial type III effector. Science 301: 1230-1233. doi:10.1126/science.1085671. PubMed: 12947197. 12947197 10.1126/science.1085671

[17] Lu D, Wu S, Gao X, Zhang Y, Shan L et al. (2010) A receptor-like cytoplasmic kinase, BIK1, associates with a flagellin receptor complex to initiate plant innate immunity. Proc Natl Acad Sci U S A 107: 496-501. doi:10.1073/pnas.0909705107. PubMed: 20018686. 20018686 10.1073/pnas.0909705107 PMC2806711

[18] Shiu SH, Bleecker AB (2001) Receptor-like kinases from Arabidopsis form a monophyletic gene family related to animal receptor kinases. Proc Natl Acad Sci U S A 98: 10763-10768. doi:10.1073/pnas.181141598. PubMed: 11526204. 11526204 10.1073/pnas.181141598 PMC58549

[19] Burr CA, Leslie ME, Orlowski SK, Chen I, Wright CE et al. (2011) CAST AWAY, a membrane-associated receptor-like kinase, inhibits organ abscission in Arabidopsis. Plant Physiol 156: 1837-1850. doi:10.1104/pp.111.175224. PubMed: 21628627. 21628627 10.1104/pp.111.175224 PMC3149937

[20] Mackey D, Holt BF, Wiig A, Dangl JL (2002) RIN4 interacts with *Pseudomonas syringae* type III effector molecules and is required for *RPM1*-mediated resistance in Arabidopsis. Cell 108: 743-754. doi:10.1016/S0092-8674(02)00661-X. PubMed: 11955429. 11955429 10.1016/s0092-8674(02)00661-x

[21] Kim MG, da Cunha L, McFall AJ, Belkhadir Y, DebRoy S et al. (2005) Two *Pseudomonas syringae* type III effectors inhibit *RIN4*-regulated basal defense in Arabidopsis. Cell 121: 749-759. doi:10.1016/j.cell.2005.03.025. PubMed: 15935761. 15935761 10.1016/j.cell.2005.03.025

[22] Chung EH, da Cunha L, Wu AJ, Gao Z, Cherkis K et al. (2011) Specific threonine phosphorylation of a host target by two unrelated type III effectors activates a host innate immune receptor in plants. Cell Host Microbe 9: 125-136. doi:10.1016/j.chom.2011.01.009. PubMed: 21320695. 21320695 10.1016/j.chom.2011.01.009 PMC3061827

[23] Liu J, Elmore JM, Fuglsang AT, Palmgren MG, Staskawicz BJ et al. (2009) RIN4 functions with plasma membrane H+-ATPases to regulate stomatal apertures during pathogen attack. PLOS Biol 7: e1000139 PubMed: 19564897. 19564897 10.1371/journal.pbio.1000139 PMC2694982

[24] Aarts N, Metz M, Holub E, Staskawicz BJ, Daniels MJ et al. (1998) Different requirements for *EDS1* and *NDR1* by disease resistance genes define at least two *R* gene-mediated signaling pathways in *Arabidopsis* . Proc Natl Acad Sci U S A 95: 10306-10311. doi:10.1073/pnas.95.17.10306. PubMed: 9707643. 9707643 10.1073/pnas.95.17.10306 PMC21504

[25] Vicente JG, Holub EB (2013) *Xanthomonas campestris* pv. *campestris* (cause of black rot of crucifers) in the genomic era is still a worldwide threat to Brassica crops. Mol Plant Pathol 14: 2-18. doi:10.1111/j.1364-3703.2012.00833.x. PubMed: 23051837. 23051837 10.1111/j.1364-3703.2012.00833.x PMC6638727

[26] Arlat M, Gough CL, Barber CE, Boucher C, Daniels MJ (1991) *Xanthomonas campestris* contains a cluster of *hrp* genes related to the larger *hrp* cluster of *Pseudomonas solanacearum* . Mol Plant Microbe Interact MPMI 4: 593-601. doi:10.1094/MPMI-4-593. PubMed: 1666525. 1666525 10.1094/mpmi-4-593

[27] Meyer D, Lauber E, Roby D, Arlat M, Kroj T (2005) Optimization of pathogenicity assays to study the *Arabidopsis thaliana-Xanthomonas campestris* pv. *campestris* pathosystem. Mol Plant Pathol 6: 327-333. doi:10.1111/j.1364-3703.2005.00287.x. PubMed: 20565661. 20565661 10.1111/j.1364-3703.2005.00287.x

[28] Xu RQ, Blanvillain S, Feng JX, Jiang BL, Li XZ et al. (2008) AvrAC(Xcc8004), a type III effector with a leucine-rich repeat domain from *Xanthomonas campestris* pathovar *campestris* confers avirulence in vascular tissues of *Arabidopsis thaliana* ecotype Col-0. J Bacteriol 190: 343-355. doi:10.1128/JB.00978-07. PubMed: 17951377. 17951377 10.1128/JB.00978-07 PMC2223733

[29] Guy E, Genissel A, Hajri A, Chabannes M, David P et al. (2013) Natural Genetic Variation of *Xanthomonas campestris* pv. campestris Pathogenicity on Arabidopsis Revealed by Association and Reverse Genetics. mBio. p. 4. 10.1128/mBio.00538-12 PMC3685212 23736288

[30] Feng F, Yang F, Rong W, Wu X, Zhang J et al. (2012) A Xanthomonas uridine 5'-monophosphate transferase inhibits plant immune kinases. Nature 485: 114-118. doi:10.1038/nature10962. PubMed: 22504181. 22504181 10.1038/nature10962

[31] Liu J, Elmore JM, Lin ZJ, Coaker G (2011) A receptor-like cytoplasmic kinase phosphorylates the host target RIN4, leading to the activation of a plant innate immune receptor. Cell Host Microbe 9: 137-146. doi:10.1016/j.chom.2011.01.010. PubMed: 21320696. 21320696 10.1016/j.chom.2011.01.010 PMC3070605

[32] Wengelnik K, Rossier O, Bonas U (1999) Mutations in the regulatory gene *hrpG* of *Xanthomonas campestris* pv. *vesicatoria* result in constitutive expression of all *hrp* genes. J Bacteriol 181: 6828-6831. PubMed: 10542187. 10542187 10.1128/jb.181.21.6828-6831.1999 PMC94150

[33] Noël L, Thieme F, Nennstiel D, Bonas U (2001) cDNA-AFLP analysis unravels a genome-wide *hrpG*-regulon in the plant pathogen *Xanthomonas campestris* pv. *vesicatoria* . Mol Microbiol 41: 1271-1281. doi:10.1046/j.1365-2958.2001.02567.x. PubMed: 11580833. 11580833 10.1046/j.1365-2958.2001.02567.x

[34] Remigi P, Anisimova M, Guidot A, Genin S, Peeters N (2011) Functional diversification of the GALA type III effector family contributes to *Ralstonia solanacearum* adaptation on different plant hosts. New Phytol 192: 976-987. doi:10.1111/j.1469-8137.2011.03854.x. PubMed: 21902695. 21902695 10.1111/j.1469-8137.2011.03854.x PMC3263339

[35] Meyer D, Cunnac S, Guéneron M, Declercq C, Van Gijsegem F et al. (2006) PopF1 and PopF2, two proteins secreted by the type III protein secretion system of *Ralstonia solanacearum*, are translocators belonging to the HrpF/NopX family. J Bacteriol 188: 4903-4917. doi:10.1128/JB.00180-06. PubMed: 16788199. 16788199 10.1128/JB.00180-06 PMC1483002

[36] Rossier O, Wengelnik K, Hahn K, Bonas U (1999) The *Xanthomonas* Hrp type III system secretes proteins from plant and mammalian bacterial pathogens. Proc Natl Acad Sci U S A 96: 9368-9373. doi:10.1073/pnas.96.16.9368. PubMed: 10430949. 10430949 10.1073/pnas.96.16.9368 PMC17789

[37] Sohn KH, Lei R, Nemri A, Jones JD (2007) The downy mildew effector proteins ATR1 and ATR13 promote disease susceptibility in *Arabidopsis thaliana* . Plant Cell 19: 4077-4090. doi:10.1105/tpc.107.054262. PubMed: 18165328. 18165328 10.1105/tpc.107.054262 PMC2217653

[38] Marino D, Froidure S, Canonne J, Ben Khaled S, Khafif M et al. (2013) Arabidopsis ubiquitin ligase MIEL1 mediates degradation of the transcription factor MYB30 weakening plant defence. Nat Communications 4: 1476. doi:10.1038/ncomms2479. PubMed: 23403577. 10.1038/ncomms2479 23403577

[39] Gyuris J, Golemis E, Chertkov H, Brent R (1993) Cdi1, a human G1 and S phase protein phosphatase that associates with Cdk2. Cell 75: 791-803. doi:10.1016/0092-8674(93)90498-F. PubMed: 8242750. 8242750 10.1016/0092-8674(93)90498-f

[40] Yarbrough ML, Li Y, Kinch LN, Grishin NV, Ball HL et al. (2009) AMPylation of Rho GTPases by *Vibrio* VopS disrupts effector binding and downstream signaling. Science 323: 269-272. doi:10.1126/science.1166382. PubMed: 19039103. 19039103 10.1126/science.1166382

[41] Hirayama T, Oka A (1992) Novel protein kinase of Arabidopsis thaliana (APK1) that phosphorylates tyrosine, serine and threonine. Plant Mol Biol 20: 653-662. doi:10.1007/BF00046450. PubMed: 1450380. 1450380 10.1007/BF00046450

[42] Afzal AJ, Wood AJ, Lightfoot DA (2008) Plant receptor-like serine threonine kinases: roles in signaling and plant defense. Mol Plant Microbe Interact MPMI 21: 507-517. doi:10.1094/MPMI-21-5-0507. PubMed: 18393610. 18393610 10.1094/MPMI-21-5-0507

[43] Veronese P, Nakagami H, Bluhm B, Abuqamar S, Chen X et al. (2006) The membrane-anchored Botrytis-INDUCED KINASE1 plays distinct roles in Arabidopsis resistance to necrotrophic and biotrophic pathogens. Plant Cell 18: 257-273. doi:10.1105/tpc.105.035576. PubMed: 16339855. 16339855 10.1105/tpc.105.035576 PMC1323497

[44] Rowland O, Ludwig AA, Merrick CJ, Baillieul F, Tracy FE et al. (2005) Functional analysis of *Avr9/Cf-9* rapidly elicited genes identifies a protein kinase, ACIK1, that is essential for full *Cf-9*-dependent disease resistance in tomato. Plant Cell 17: 295-310. doi:10.1105/tpc.104.026013. PubMed: 15598806. 15598806 10.1105/tpc.104.026013 PMC544506

[45] Kinch LN, Yarbrough ML, Orth K, Grishin NV (2009) Fido, a novel AMPylation domain common to fic, doc, and AvrB. PLOS ONE 4: e5818. doi:10.1371/journal.pone.0005818. PubMed: 19503829. 19503829 10.1371/journal.pone.0005818 PMC2686095

[46] Al-Daoude A, de Torres Zabala M, Ko JH, Grant M (2005) RIN13 is a positive regulator of the plant disease resistance protein RPM1. Plant Cell 17: 1016-1028. doi:10.1105/tpc.104.028720. PubMed: 15722472. 15722472 10.1105/tpc.104.028720 PMC1069715

[47] Eitas TK, Nimchuk ZL, Dangl JL (2008) Arabidopsis TAO1 is a TIR-NB-LRR protein that contributes to disease resistance induced by the *Pseudomonas syringae* effector AvrB. Proc Natl Acad Sci U S A 105: 6475-6480. doi:10.1073/pnas.0802157105. PubMed: 18424557. 18424557 10.1073/pnas.0802157105 PMC2327211

[48] Stael S, Bayer RG, Mehlmer N, Teige M (2011) Protein N-acylation overrides differing targeting signals. FEBS Lett 585: 517-522. doi:10.1016/j.febslet.2011.01.001. PubMed: 21219905. 21219905 10.1016/j.febslet.2011.01.001 PMC3971372

[49] Elmore JM, Liu J, Smith B, Phinney B, Coaker G (2012) Quantitative proteomics reveals dynamic changes in the plasma membrane during Arabidopsis immune signaling. Molecular Cell Proteomics MCP 11: 014555 PubMed: 22215637. 10.1074/mcp.M111.014555 PMC3322570 22215637

[50] Afzal AJ, da Cunha L, Mackey D (2011) Separable fragments and membrane tethering of Arabidopsis RIN4 regulate its suppression of PAMP-triggered immunity. Plant Cell 23: 3798-3811. doi:10.1105/tpc.111.088708. PubMed: 21984695. 21984695 10.1105/tpc.111.088708 PMC3229150

[51] van der Hoorn RA, Kamoun S (2008) From Guard to Decoy: a new model for perception of plant pathogen effectors. Plant Cell 20: 2009-2017. doi:10.1105/tpc.108.060194. PubMed: 18723576. 18723576 10.1105/tpc.108.060194 PMC2553620

[52] Blanvillain S, Meyer D, Boulanger A, Lautier M, Guynet C et al. (2007) Plant carbohydrate scavenging through TonB-dependent receptors: a feature shared by phytopathogenic and aquatic bacteria. PLOS ONE 2: e224. doi:10.1371/journal.pone.0000224. PubMed: 17311090. 17311090 10.1371/journal.pone.0000224 PMC1790865

[53] Boucher CA, Barberis P, Trigalet AP, Demery DA (1985) Transposon mutagenesis of *Pseudomonas solanacearum*: isolation of Tn*5*-induced avirulent mutants. J Gen Microbiol 131: 2449-2457.

[54] King EO, Ward MK, Raney DE (1954) Two simple media for the demonstration of pyocyanin and fluorescin. J Lab Clin Med 44: 301-307. PubMed: 13184240. 13184240

[55] Yu GL, Katagiri F, Ausubel FM (1993) *Arabidopsis* mutations at the *RPS2* locus result in loss of resistance to *Pseudomonas syringae* strains expressing the avirulence gene *avrRpt2* . MPMI 6: 434-443. doi:10.1094/MPMI-6-434. PubMed: 8400373. 8400373 10.1094/mpmi-6-434

[56] Mackey D, Belkhadir Y, Alonso JM, Ecker JR, Dangl JL (2003) *Arabidopsis* RIN4 is a target of the type III virulence effector AvrRpt2 and modulates *RPS2*-mediated resistance. Cell 112: 379-389. doi:10.1016/S0092-8674(03)00040-0. PubMed: 12581527. 12581527 10.1016/s0092-8674(03)00040-0

[57] Grant MR, Godiard L, Straube E, Ashfield T, Lewald J et al. (1995) Structure of the *Arabidopsis RPM1* gene enabling dual specificity disease resistance. Science 269: 843-846. doi:10.1126/science.7638602. PubMed: 7638602. 7638602 10.1126/science.7638602

[58] Warren RF, Merritt PM, Holub E, Innes RW (1999) Identification of three putative signal transduction genes involved in *R* gene-specified diseases resistance in Arabidopsis. Genetics 152: 1-12. PubMed: 10224239. 10224270 10.1093/genetics/152.1.401 PMC1460583

[59] Deslandes L, Pileur F, Liaubet L, Camut S, Can C et al. (1998) Genetic characterization of *RRS1*, a recessive locus in *Arabidopsis thaliana* that confers resistance to the bacterial soilborne pathogen *Ralstonia solanacearum* . Mol Plant Microbe Interact MPMI 11: 659-667. doi:10.1094/MPMI.1998.11.7.659. PubMed: 9650298. 9650298 10.1094/MPMI.1998.11.7.659

[60] Schäfer A, Tauch A, Jäger W, Kalinowski J, Thierbach G et al. (1994) Small mobilizable multi-purpose cloning vectors derived from the *Escherichia coli* plasmids pK18 and pK19: selection of defined deletions in the chromosome of *Corynebacterium glutamicum* . Gene 145: 69-73. doi:10.1016/0378-1119(94)90324-7. PubMed: 8045426. 8045426 10.1016/0378-1119(94)90324-7

[61] Engler C, Kandzia R, Marillonnet S (2008) A one pot, one step, precision cloning method with high throughput capability. PLOS ONE 3: e3647. doi:10.1371/journal.pone.0003647. PubMed: 18985154. 18985154 10.1371/journal.pone.0003647 PMC2574415

[62] Ditta G, Stanfield S, Corbin D, Helinski DR (1980) Broad host range DNA cloning system for Gram-negative bacteria: Construction of a gene bank of *Rhizobium meliloti* . Proc Natl Acad Sci U S A 77: 7347-7351. doi:10.1073/pnas.77.12.7347. PubMed: 7012838. 7012838 10.1073/pnas.77.12.7347 PMC350500

[63] Figurski DH, Helinski DR (1979) Replication of an origin-containing derivative of plasmid RK2 dependent on a plasmid function provided in *trans* . Proc Natl Acad Sci U S A 76: 1648-1652. doi:10.1073/pnas.76.4.1648. PubMed: 377280. 377280 10.1073/pnas.76.4.1648 PMC383447

[64] Cunnac S, Occhialini A, Barberis P, Boucher C, Genin S (2004) Inventory and functional analysis of the large Hrp regulon in *Ralstonia solanacearum*: identification of novel effector proteins translocated to plant host cells through the type III secretion system. Mol Microbiol 53: 115-128. doi:10.1111/j.1365-2958.2004.04118.x. PubMed: 15225308. 15225308 10.1111/j.1365-2958.2004.04118.x

[65] Rivas S, Rougon-Cardoso A, Smoker M, Schauser L, Yoshioka H et al. (2004) CITRX thioredoxin interacts with the tomato Cf-9 resistance protein and negatively regulates defence. EMBO J 23: 2156-2165. doi:10.1038/sj.emboj.7600224. PubMed: 15131698. 15131698 10.1038/sj.emboj.7600224 PMC424418

[66] Gietz RD, Woods RA (2001) Genetic transformation of yeast. BioTechniques 30: 816-820, 822-816, 828 passim 10.2144/01304rv02 11314265

[67] Caspari T, Dahlen M, Kanter-Smoler G, Lindsay HD, Hofmann K et al. (2000) Characterization of *Schizosaccharomyces pombe* Hus1: a PCNA-related protein that associates with Rad1 and Rad9. Mol Cell Biol 20: 1254-1262. doi:10.1128/MCB.20.4.1254-1262.2000. PubMed: 10648611. 10648611 10.1128/mcb.20.4.1254-1262.2000 PMC85258

[68] Witte CP, Noël LD, Gielbert J, Parker JE, Romeis T (2004) Rapid one-step protein purification from plant material using the eight-amino acid StrepII epitope. Plant Mol Biol 55: 135-147. doi:10.1007/s11103-004-0501-y. PubMed: 15604670. 15604670 10.1007/s11103-004-0501-y

