# Supplementary material for: xopAC-triggered Immunity against Xanthomonas Depends on Arabidopsis Receptor-Like Cytoplasmic Kinase Genes PBL2 and RIPK
Source: PLoS One. 2013 Aug 9;8(8):e73469. doi: 10.1371/journal.pone.0073469 (PMC3739749; doi:10.1371/journal.pone.0073469)
Supplement: Figure S1 — The LRR and fic domains of XopAC are not required for pathogenicity on Arabidopsis ecotype Kas. (PDF) [file pone.0073469.s001.pdf]

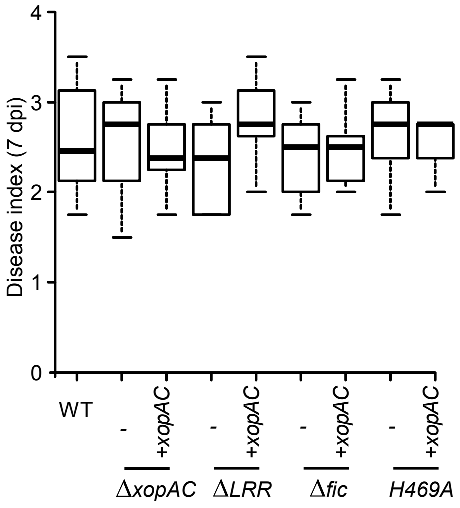

Supporting Figure S1. The LRR and fic domains of XopAC are not required for pathogenicity on Arabidopsis ecotype Kas.

A boxplot representation of pathogenicity of wild-type *Xcc* strain 8004, *xopAC* mutants *(Δ xopAC, ΔLRR, Δfic, xopAC*-H469A) and their complemented derivatives with pCZ917-xopAC$_A$ (+xopAC) is shown: middle bar = median; box limit = upper and lower quartile; extremes = Min and Max values. Bacteria were inoculated by piercing the central vein and infection symptoms scored 7 days post-inoculation. Disease index indicates: 0-1 no symptoms; 1-2 weak chlorosis, 2-3 strong chlorosis; 3-4 necrosis. N=3. Each time at least 4 plants were inoculated on at least 3 leaves. No significant differences to the wild type could be observed (Tukey HSD test; P<0.01).
