# Supplementary material for: xopAC-triggered Immunity against Xanthomonas Depends on Arabidopsis Receptor-Like Cytoplasmic Kinase Genes PBL2 and RIPK
Source: PLoS One. 2013 Aug 9;8(8):e73469. doi: 10.1371/journal.pone.0073469 (PMC3739749; doi:10.1371/journal.pone.0073469)
Supplement: Figure S2 — Accumulation and stability of XopAC variants expressed in Xcc 8004*, Pst DC3000 and N . benthamiana . (PDF) [file pone.0073469.s002.pdf]

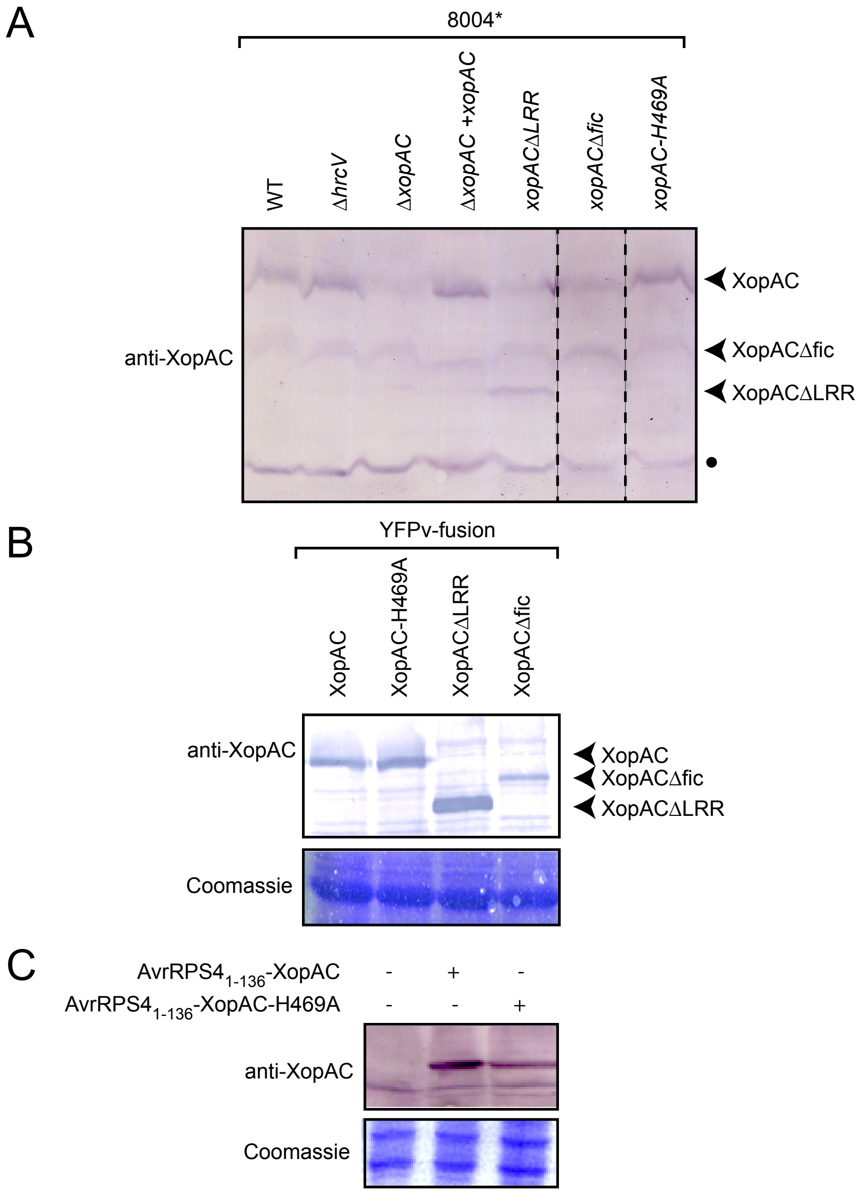

Supporting Figure S2: Accumulation and stability of XopAC variants expressed in *Xcc* 8004*, *Pst* DC3000 and *N. benthamiana*.

Total protein extracts were separated by SDS-PAGE and analysed by immunoblot using the anti-XopAC antibody. (A) Derivatives of *Xcc* strain 8004* (*hrpG**) expressing *xopAC* variants grown in Moka medium. Black dot indicates an unspecific band used to compare loading between lanes. (B) YFPv-XopAC fusions expressed in *N. benthamiana* leaves using *Agrobacterium*-mediated transformation. Arrows indicate the position of full-length XopAC and its ΔLRR and Δfic variants. (C) *Pst* DC3000 expressing XopAC fusions to the AvrRps4 N terminus. Coomassie stained portions of the gel are shown to compare sample loading between lanes.
