# Supplementary material for: xopAC-triggered Immunity against Xanthomonas Depends on Arabidopsis Receptor-Like Cytoplasmic Kinase Genes PBL2 and RIPK
Source: PLoS One. 2013 Aug 9;8(8):e73469. doi: 10.1371/journal.pone.0073469 (PMC3739749; doi:10.1371/journal.pone.0073469)
Supplement: Figure S3 — Symptom development and in planta growth of Xcc strain 8004 infiltrated into leaves of Arabidopsis ecotype Kas are not affected by XopAC. (PDF) [file pone.0073469.s003.pdf]

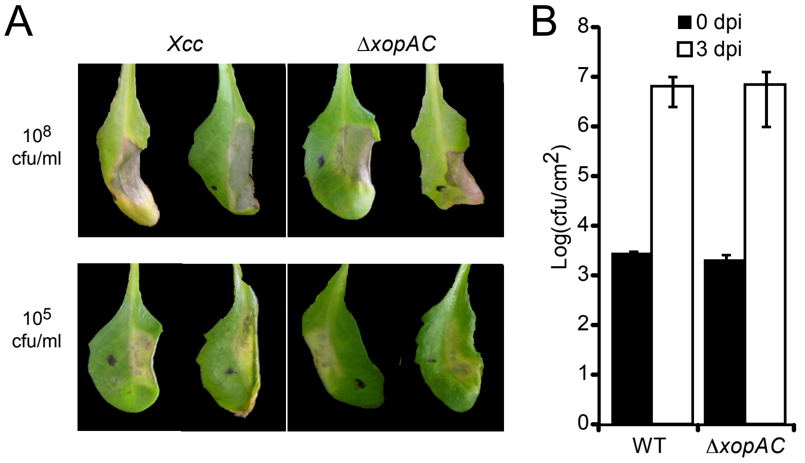

Supporting Figure S3. Symptom development and *in planta* growth of *Xcc* strain 8004 infiltrated into leaves of Arabidopsis ecotype Kas are not affected by XopAC.

(A) Wild-type *Xcc* strain 8004 and 8004Δ*xopAC* were force-infiltrated at bacterial densities of 10^5 and 10^8 cfu/ml in four-weeks-old Arabidopsis plants ecoytpe Kas. Pictures were taken at 5 days post-inoculation. (B) *Xcc* strains were infiltrated into leaves of Arabidopsis ecotype Kas at a bacterial density of 10^5 cfu/ml. *In planta* bacterial populations in the inoculated areas were determined 0 and 3 days post-inoculation (dpi) and expressed as log (cfu/cm^2). Standard deviations were calculated on three independent experiments: for each experiment, three samples of two leaf discs from different plants per strain. No statistically significant groups could be identified using a Wilcoxon test (P<0.05).
