# Supplementary material for: xopAC-triggered Immunity against Xanthomonas Depends on Arabidopsis Receptor-Like Cytoplasmic Kinase Genes PBL2 and RIPK
Source: PLoS One. 2013 Aug 9;8(8):e73469. doi: 10.1371/journal.pone.0073469 (PMC3739749; doi:10.1371/journal.pone.0073469)
Supplement: Figure S5 — XopAC-H469A interacts with the PIX8 kinase domain in a yeast two-hybrid assay. (PDF) [file pone.0073469.s005.pdf]

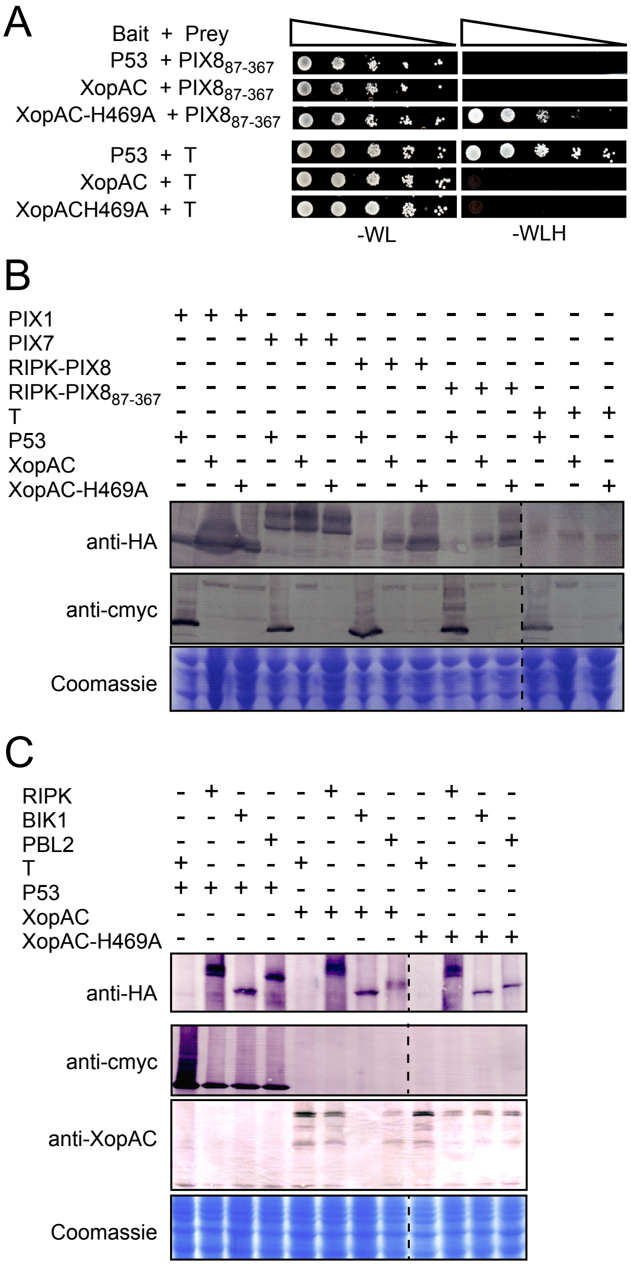


**Supporting Figure S5. XopAC-H469A interacts with the PIX8 kinase domain in a yeast two-hybrid assay.**

(A) Yeast two-hybrid interaction test between XopAC or its mutant allele H469A as baits and $PIX8_{87-367}$ and T antigen as preys. The T antigen is a known interactor of P53 and was used as specificity control for the baits. Ten-fold serial dilutions of yeast transformants were spotted from left to the right on minimal medium (-WL) and medium without histidine (-WLH) which is used to visualize prey/bait interaction. Pictures were taken 4 days after spotting. (B, C) Accumulation of bait and prey proteins described in panel A and Figure 4 was verified by immunoblot analysis of total yeast protein extracts using the anti-HA, anti-XopAC and anti-cmyc antibodies. Equal protein loading was tested by coomassie staining of the SDS-PAGE.
