# Supplementary material for: xopAC-triggered Immunity against Xanthomonas Depends on Arabidopsis Receptor-Like Cytoplasmic Kinase Genes PBL2 and RIPK
Source: PLoS One. 2013 Aug 9;8(8):e73469. doi: 10.1371/journal.pone.0073469 (PMC3739749; doi:10.1371/journal.pone.0073469)
Supplement: Figure S6 — Pathogenicity and in planta growth of strain 8004∆xopAC on Col-0 mutants and transgenics inoculated by piercing the central leaf vein. (PDF) [file pone.0073469.s006.pdf]

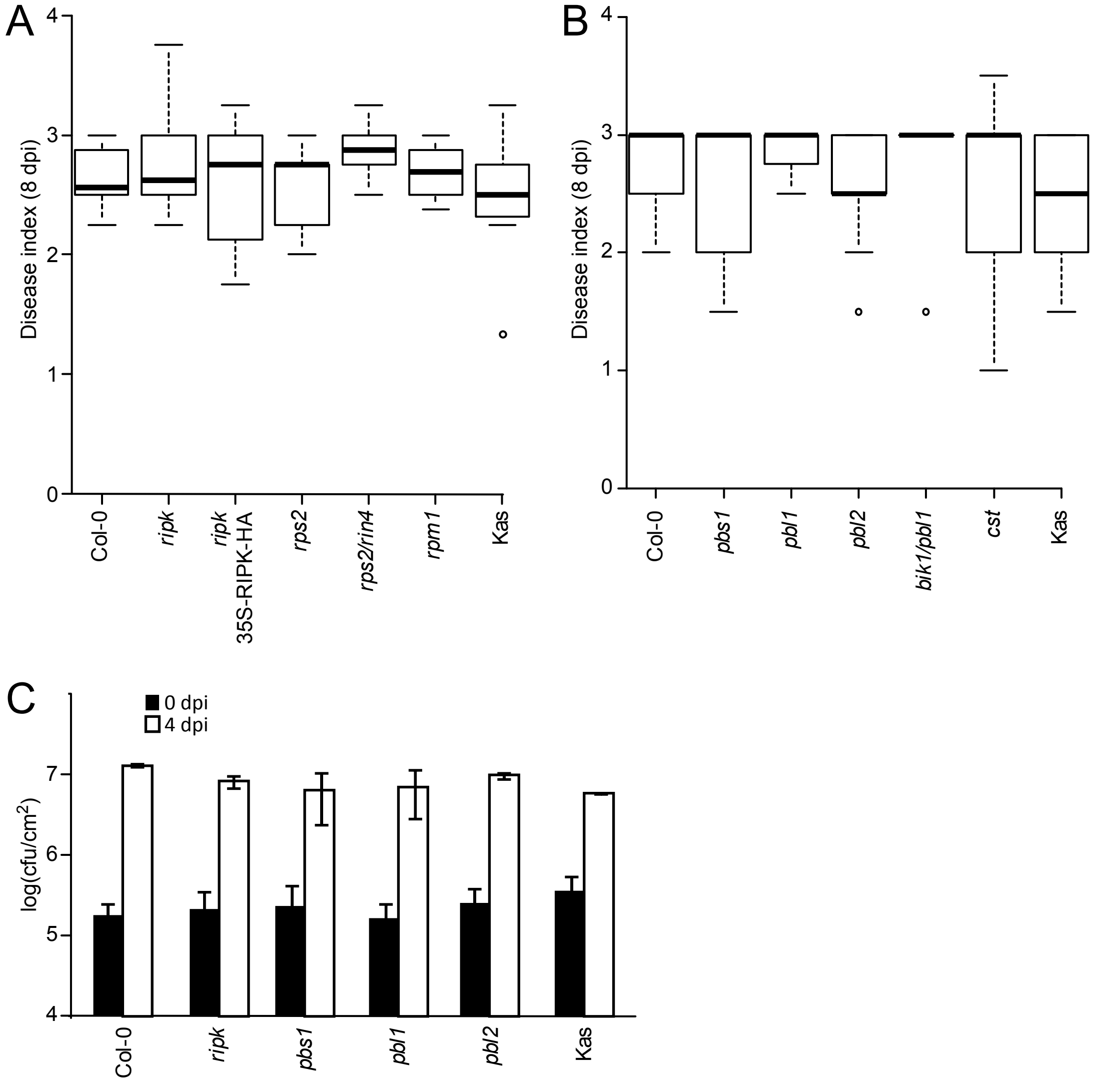

Supporting Figure S6. Pathogenicity and *in planta* growth of strain 8004Δ*xopAC* on Col-0 mutants and transgenics inoculated by piercing in the central leaf vein.

A boxplot representation of disease index scores is shown: middle bar = median; box limit = upper and lower quartile; extremes = Min and Max values. Kas was used as a susceptible control. Mutants in genes coding for the RIPK/RIN4/RPM1 complex (A) and other RLCK (B) were tested. Disease indexes were scored 8 days post-inoculation: 0-1 no symptoms; 1-2 weak chlorosis, 2-3 strong chlorosis; 3-4 necrosis. N=3. Each time at least 4 plants were inoculated on at least 3 leaves. Statistical groups were determined using a Tukey HSD test ($P<0.001$) and indicated by a letter. (C) A bacterial suspension (10^5 cfu/ml) of *Xcc* strain 8004Δ*xopAC* was inoculated by piercing leaves of Col-0 mutants and transgenics. *In planta* bacterial populations in the inoculated areas were determined 0 and 4 days post-inoculation and expressed as log (cfu/cm^2). Standard deviations were calculated on two independent experiments: for each experiment, three samples of two leaf disc from different plants per strain. No statistically significant groups could be identified using a Wilcoxon test ($P<0.05$).
