# Supplementary material for: xopAC-triggered Immunity against Xanthomonas Depends on Arabidopsis Receptor-Like Cytoplasmic Kinase Genes PBL2 and RIPK
Source: PLoS One. 2013 Aug 9;8(8):e73469. doi: 10.1371/journal.pone.0073469 (PMC3739749; doi:10.1371/journal.pone.0073469)
Supplement: Table S1 — Strains and plasmids used in this study. (PDF) [file pone.0073469.s007.pdf]

Supporting Table S1: Strains and plasmids used in this study

Strains and plasmids	Features	Reference or source
Xanthomonas campestris pv. *campestris*		
8004	Wild-type strain, Rif^r	[1]
8004Δ*xopAC*	8004 derivative, carries a *xopAC* deletion, Rif^r	M. Chabannes, unpublished
8004*xopAC*Δ*LRR*	8004 derivative, carries a deletion of XopAC LRR domain, Rif^r	This study
8004*xopAC*Δ*Fic*	8004 derivative, carries a deletion of XopAC Fic domain, Rif^r	This study
8004*xopAC-H469A*	8004 derivative, carries the XopAC H469A mutation, Rif^r	This study
8004*	8004 derivative, carries a *hrpG** mutation; constitutive expression of *hrpG* regulon, Rif^r	This study
8004**hrcV*	8004* derivative, carries a *hrcV*deletion, Rif^r	This study
Ralstonia solanacearum		
GMI1000	Wild-type strain	[2]
GMI1694	GMI1000 *hrcV*::Ω, Spc^r	[3]
Pseudomonas syringae pv. *tomato*		
DC3000	Wild-type strain, Rif^r	[4]
Agrobacterium tumefaciens		
C58C1	Wild-type strain, Rif^r	-
GV3101 pMP90	Wild-type, Rif^r, Gent^r	-
Escherichia coli		
TG1	*supE thi-1* Δ*(lac-proAB)* Δ*(mcrB-hsdSM)5* $(r_k^- m_k^-)$ *[F′ traD36 proAB lacI*^{q}*Z*Δ*M15]*	Stratagene
DH5α	*F*$^-$ *recA lacZ DM15*	Bethesda Research Laboratory
BL21 (DE3) pLysS	F- *ompT hsdS*$_B$ ($r_B^- m_B^-$) *gal dcm* (DE3) pLysS, Cm^r	Invitrogen
Saccharomyces cerevisiae		
AH109	*MATa, trp1-901, leu2-3, 112, ura3-52, his3-200, gal4*Δ*, gal80*Δ*, LYS2 : : GAL1*$_{UAS}$*-GAL1*$_{TATA}$*-HIS3, GAL2*$_{UAS}$*-GAL2*$_{TATA}$*-ADE2, URA3 : : MEL1*$_{UAS}$*-MEL1* $_{TATA}$*-lacZ*	Clontech
NMY32	MATa his3Δ200 trp1-901 leu2-3,112 (lexAop)$_8$-ADE2 LYS2::(lexAop)$_4$-HIS3 URA3::(lexAop)$_8$-lacZ GAL4)	Dual Systems Biotech
Plasmids		
pRK2073	Helper plasmid, Spc^r, Strep^r	[5]
pCZ917	pFAJ1700 derivative with the pTaq Promoter and T7 Terminator, *lacI*,Tet^r,	[6]
pCZ917-xopAC$_A$	pCZ917 with the *xopAC*$_{8004}$ promoter and gene, Tet^r	M. Chabannes, unpublished
pΔ13	Goldengate cloning-compatible derivative of suicide plasmid pK18mobsacB, Kan^r, Suc^s	L. Noël, unpublished
pΔ13-xopACΔLRR	pK18mobsacB derivative, for the deletion of *xopAC*$_{8004}$ LRR, Kan^r, Suc^s	This study
pΔ13-xopACΔfic	pK18mobsacB derivative, for the deletion of *xopAC*$_{8004}$ fic, Kan^r, Suc^s	This study

pΔ13-xopAC-H469A	pΔ13 derivative, for $XopAC_{8004}$ H469A substitution, Kan^r, Suc^s	This study
pΔ13-hrpG*	pK18mobsacB derivative, for $HrpG_{8004}$ E44K substitution, Kan^r, Suc^s	This study
pDONR207	Gateway Donor vector for entry clone construction by BP recombination, $Gent^r$, Cm^r	Invitrogen
pENTRY-xopAC	pDONR207 derivative, $XopAC_{8004}$ ORF cloned by BP reaction, $Gent^r$	This study
pENTRY-xopACΔLRR	pDONR207 derivative, $XopAC\Delta LRR_{8004}$ ORF cloned by BP reaction, $Gent^r$	This study
pENTRY-xopACΔfic	pDONR207 derivative, $XopAC\Delta fic_{8004}$ ORF cloned by BP reaction, $Gent^r$	This study
pENTRY-xopAC-H469A	pDONR207 derivative, $XopAC\text{-}H469A_{8004}$ ORF cloned by BP reaction, $Gent^r$	This study
pENTRY-PIX1	pDONR207 derivative, PIX1 ORF cloned by BP reaction, $Gent^r$	This study
pENTRY-PIX7	pDONR207 derivative, PIX7 ORF cloned by BP reaction, $Gent^r$	This study
pENTRY-PIX8	pDONR207 derivative, PIX8 ORF cloned by BP reaction, $Gent^r$	This study
pENTRY-$PIX8_{87\text{-}367}$	pDONR207 derivative, $PIX8_{87\text{-}367}$ ORF cloned by BP reaction, $Gent^r$	This study
pDEST17	His-GW, Amp^r, Cm^r	Invitrogen
pDEST17-xopAC	IPTG-inducible expression of His6-XopAC, Amp^r	This study
pGADT7-GW	Gateway-compatible prey vector Gal4-AD-HA-GW, Amp^r, Cm^r, Leu^+	L. Deslandes, unpublished
pGADT7-PIX1	Prey vector Gal4-AD-HA-PIX1, Amp^r, Leu^+	This study
pGADT7-PIX7	Prey vector Gal4-AD-HA-PIX7, Amp^r, Leu^+	This study
pGADT7-PIX8	Prey vector Gal4-AD-HA-PIX8, Amp^r, Leu^+	This study
pGADT7-$PIX8_{87\text{-}367}$	Prey vector Gal4-AD-HA-$PIX8_{87\text{-}367}$, Amp^r, Leu^+	This study
pGBKT7-GW	Gateway-compatible bait vector, Gal4-BD-myc-GW, Kan^r, Cm^r, Trp^+	L. Deslandes, unpublished
pGBKT7-xopAC	Gateway-compatible bait vector, Gal4-BD-myc-XopAC, Kan^r, Trp^+	This study
pGBKT7-xopAC-H469A	Gateway-compatible bait vector, Gal4-BD-myc-XopAC-H469A, Kan^r, Trp^+	This study
pEDV6	pAvrRPS4::$AvrRPS4_{1\text{-}136}$-GW-HA, Gen^r, Cm^r	[7]
pEDV6-xopAC	pEDV6 derivative, pAvrRPS4::$AvrRPS4_{1\text{-}136}$-$XopAC_{8004}$-HA fusion, Gen^r	This study
pEDV6-xopAC-H469A	pEDV6 derivative, pAvrRPS4::$AvrRPS4_{1\text{-}136}$-$XopAC_{8004}$-H469A-HA fusion, Gen^r	This study
pNP269	pRC derivative [8], pGALA7-GW-HA, Amp^r, Kan^r, Gen^r, Cm^r	N. Peeters, unpublished
pNP269-xopAC	pNP269 derivative, pGALA7-XopAC-HA, Amp^r, Kan^r, Gen^r	This study
pNP269-xopAC-H469A	pNP269 derivative, pGALA7-XopAC-H469A-HA, Amp^r, Kan^r, Gen^r	This study
pBin19-35S-YFPv-GW	p35S::YFPv-GW, Tet^r, Kan^r, Cm^r	S. Rivas, unpublished
pBin19-YFPv-xopAC	p35S::YFPv-xopAC, Tet^r, Kan^r	This study
pBin19-YFPv-xopACΔLRR	p35S::YFPv-xopACΔLRR, Tet^r, Kan^r	This study
pBin19-YFPv-xopACΔfic	p35S::YFPv-xopACΔfic, Tet^r, Kan^r	This study
pBin19-YFPv-xopAC-H469A	p35S::YFPv-xopAC-H469A, Tet^r, Kan^r	This study

pBin19-MIEL1-CFP	p35S:: MIEL1-CFP	S. Rivas, unpublished

References

1. Daniels MJ, Barber CE, Turner PC, Sawczyc MK, Byrde RJ, et al. (1984) Cloning of genes involved in pathogenicity of *Xanthomonas campestris* pv. *campestris* using the broad host range cosmid pLAFR1. The EMBO journal 3: 3323-3328.
2. Boucher CA, Van Gijsegem F, Barberis PA, Arlat M, Zischek C (1987) *Pseudomonas solanacearum* genes controlling both pathogenicity on tomato and hypersensitivity on tobacco are clustered. Journal of bacteriology 169: 5626-5632.
3. Cunnac S, Occhialini A, Barberis P, Boucher C, Genin S (2004) Inventory and functional analysis of the large Hrp regulon in *Ralstonia solanacearum*: identification of novel effector proteins translocated to plant host cells through the type III secretion system. Molecular microbiology 53: 115-128.
4. Cuppels DA (1986) Generation and Characterization of Tn*5* Insertion Mutations in *Pseudomonas syringae* pv. *tomato*. Applied and environmental microbiology 51: 323-327.
5. Leong SA, Ditta GS, Helinski DR (1982) Heme biosynthesis in *Rhizobium*. Identification of a cloned gene coding for delta-aminolevulinic acid synthetase from *Rhizobium meliloti*. The Journal of biological chemistry 257: 8724-8730.
6. Boulanger A, Dejean G, Lautier M, Glories M, Zischek C, et al. (2010) Identification and regulation of the N-acetylglucosamine utilization pathway of the plant pathogenic bacterium *Xanthomonas campestris* pv. *campestris*. Journal of bacteriology 192: 1487-1497.
7. Fabro G, Steinbrenner J, Coates M, Ishaque N, Baxter L, et al. (2011) Multiple candidate effectors from the oomycete pathogen *Hyaloperonospora arabidopsidis* suppress host plant immunity. PLoS pathogens 7: e1002348.
8. Monteiro F, Sole M, van Dijk I, Valls M (2012) A chromosomal insertion toolbox for promoter probing, mutant complementation, and pathogenicity studies in *Ralstonia solanacearum*. Molecular plant-microbe interactions : MPMI 25: 557-568.
