# Supplementary material for: xopAC-triggered Immunity against Xanthomonas Depends on Arabidopsis Receptor-Like Cytoplasmic Kinase Genes PBL2 and RIPK
Source: PLoS One. 2013 Aug 9;8(8):e73469. doi: 10.1371/journal.pone.0073469 (PMC3739749; doi:10.1371/journal.pone.0073469)
Supplement: Table S2 — Oligonucleotides used in this study. (PDF) [file pone.0073469.s008.pdf]

Supporting Table S2: Oligonucleotides used in this study.

Name	Sequence (5'-3')[a]
LN202	CTTTTCAGACGAACTCAAATTGCTGCGCTGCCTG
LN203	CAGGCAGCGCAGCAATTTGAGTTCGTCTGAAAAG
LN251	TTTGGTCTCAAGGTGATCATGCCGGGCTCTCTGAAGG
LN252	TTTGGTCTCACTTGCAGCTCAGCAAGCTGCGGTGCGATTG
LN433	AGCGCTTGGTCAGTCTGGCTCCTTTCGATGACGCAAATG
LN434	CATTTGCGTCATCGAAAGGAGCCAGACTGACCAAGCGCT
LN436	TTTGGTCTCAAGGTCAGAGGTTGTCCTACCAAGCTTCAG
LN439	TTTGGTCTCATTTCGGGCGGAGGGTACCGACACTC
LN442	TTTGGTCTCACTTGCAGCCGAATCGGCGACGCGAAAGGTG
LN443	TTTGGTCTCAGAAACCCTGAAAGACGAAATGGCGAAG
LN608	AACTGCAGATGGATAAAAATCTTAATTTGTG
LN609	AACCCGGGCTACTGGTGAACCTGGTTCATAAC
LN714	GGGGACAAGTTTGTACAAAAAAGCAGGCTTCATGGGTTCTTGCTTCAGTTCTC
LN715	GGGGACCACTTTGTACAAGAAAGCTGGGTCCACAAGGTGCCTGCCAAAAGG
LN716	GGGGACAAGTTTGTACAAAAAAGCAGGCTTCATGGGTAATTGTTTAGATTCATCAG
LN717	GGGGACCACTTTGTACAAGAAAGCTGGGTCTCTTACACGAGGAGATTGAGTG
MC4	AGCTTGGTAGGACAAAGCTTGTCCCGCATCTAAGCA
MC5	GATGCGGGACAAGCTTTGTCCTACCAAGCTTCAGCGCT
MC8	TTCACCAACGGGCGGCCGATCAAGTGTGATCGGTGC
MC9	ATCACACTTGATCGGCCGCCCGTTGGTGAAGCAAGC
MC12	AAAAAGCAGGCTCGATGGATAAAAATCTTAATTT
MC13	AGAAAGCTGGGTAGCATTGAGGCGTGTAGGT
MC18	GGGGACAAGTTTGTACAAAAAAGCAGGCT
MC19	GGGGACCACTTTGTACAAGAAAGCTGGGT
EG3	CGACGTTCGATCCAGAAACCAGACAGAGGTTG
EG4	CAACCTCTGTCTGGTTTCTGGATCGAACGTCG
EG5	TTTGGTCTCAAGGTTGGATAAAAATCTTAATTTGTGGG
EG6	TTTGGTCTCACTTGGGGCGGAGGGTACCGACACTCCG
EG7	TTTGGTCTCAAGGTATTGGCCAATGCAGCAGCCTGACTACG
EG8	TTTGGTCTCACTTGCCTGAATCACCACCAGGCCGCCGC
EG45	GGGACAAGTTTGTACAAAAAAGCAGGCTTCATGAGAGACTCTTCAACAACTGC
EG46	GGGGACCACTTTGTACAAGAAAGCTGGGTTCACGATCTTAACGAACCCGGAGC
EG58	GGGACAAGTTTGTACAAAAAAGCAGGCTTCATGGGTTTGGATGCTGTTAAAGC
EG59	GGGGACCACTTTGTACAAGAAAGCTGGGTCTATGTAGTTGCTCCTTTAGGC
EG62	GGGACAAGTTTGTACAAAAAAGCAGGCTTCATGGCGGTGAAGAAGAAAGTTTCATGG
EG63	GGGACCACTTTGTACAAGAAAGCTGGGTTTAGTACCGTTCCCCACCTGCC
EG167	GGGACAAGTTTGTACAAAAAAGCAGGCTTCTTTTCATCGACCAATTTTCTA
EG168	GGGACCACTTTGTACAAGAAAGCTGGGTTCAAAGATCATTGAGTATAGAGAC

[a] underlined sequences indicate restriction or *att* sites used for cloning purposes.
