# Supplementary material for: xopAC-triggered Immunity against Xanthomonas Depends on Arabidopsis Receptor-Like Cytoplasmic Kinase Genes PBL2 and RIPK
Source: PLoS One. 2013 Aug 9;8(8):e73469. doi: 10.1371/journal.pone.0073469 (PMC3739749; doi:10.1371/journal.pone.0073469)
Supplement: Figure S4 — Protein alignment of the eight full-length PIX proteins belonging to the RLCK family identified by yeast two-hybrid assay as putative interactors with XopAC-H469A. (PDF) [file pone.0073469.s004.pdf]

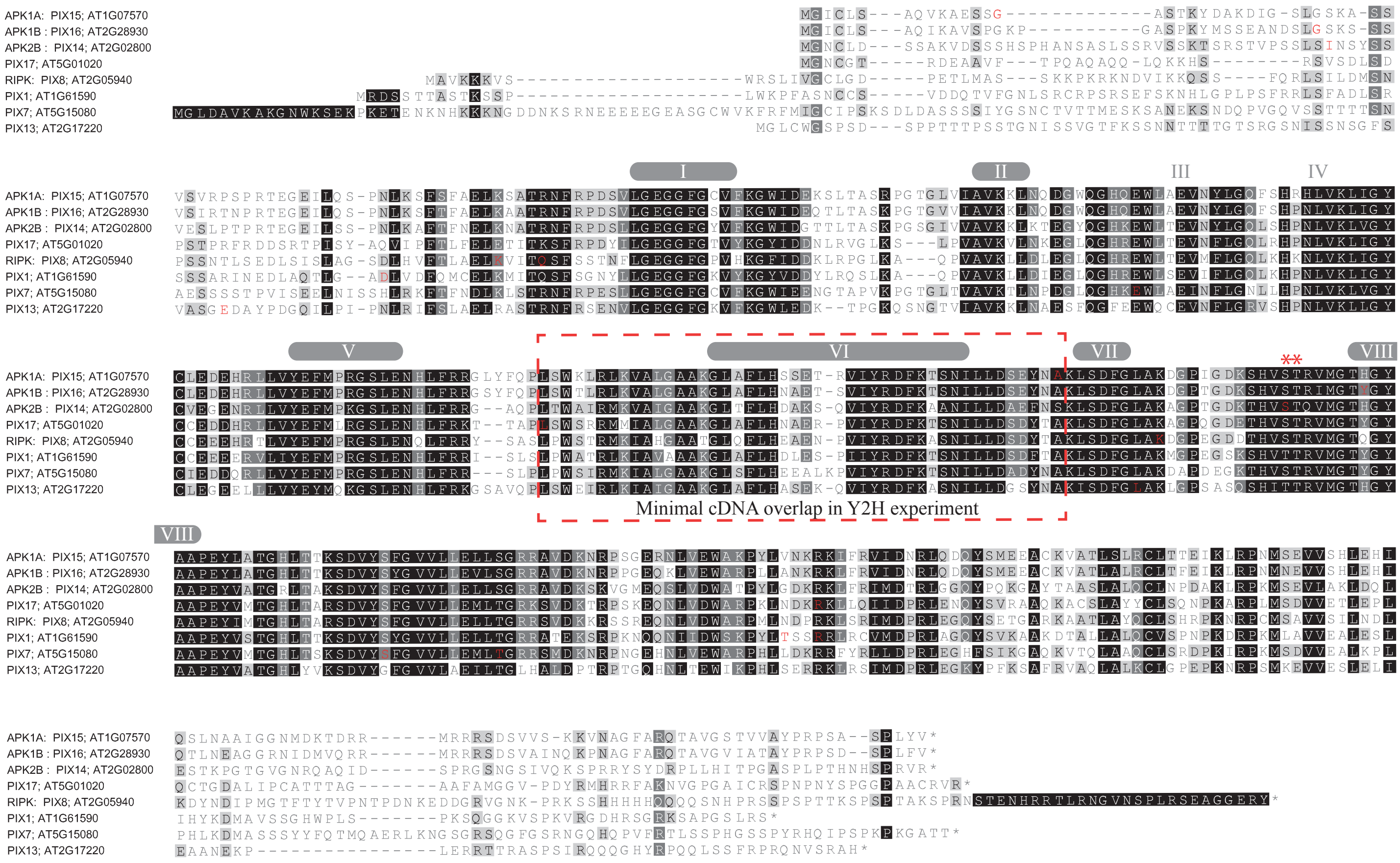

Supporting Figure S4. Protein alignment of the eight full-length PIX proteins belonging to the RLCK family identified by yeast two-hybrid assay as putative interactors with XopAC-H469A.

Alignment was performed using Geneious aligment default parameters. Black, grey and light grey highlighting of amino acids indicates, 100%, 80-100% and 60-80% of similarity, respectively. The two red asterisks indicate the Serine-Threonine residues of RIPK/PIX8 uridylylated by XopAC (Feng *et al.*, 2012). Amino acids corresponding to the borders of the cDNA clones identified by yeast two-hybrid assay are highlighted in red. A red frame represents the minimal overlap between these 11 different cDNA clones. The 8 conserved kinase domains are indicated with roman numerals.
